# A category theory perspective on the Language of Thought: LoT is universal

**DOI:** 10.3389/fpsyg.2024.1361580

**Published:** 2024-04-30

**Authors:** Steven Phillips

**Affiliations:** Mathematical Neuroscience Group, Human Informatics and Interaction Research Institute, National Institute of Advanced Industrial Science and Technology, Tsukuba, Japan

**Keywords:** Language of Thought, category theory, topos theory, category, topos, product, subobject

## Abstract

The Language of Thought (LoT) hypothesis proposes that some collections of mental states and processes are symbol systems to explain language-like systematic properties of thought. Recent proponents of this hypothesis point to additional LoT-like properties in non-linguistic domains to claim that LoT remains the “best game in town” in terms of explanatory coverage. Nonetheless, LoT assumes but does not explain why/how symbolic representations connect to other (non-symbolic) formats. The perspective presented here is supposed to bridge this gap as a duality in a category theoretical sense: (perceptual) data are projected onto a base (conceptual) space in one direction, and in the opposite direction, these data are referenced by that space. Accordingly, perception is dual to conception. These constructions follow from a *universal mapping principle* affording an explanation for why/how symbolic and non-symbolic formats are connected: as the “best” possible transformation between the two forms— so the slogan, *LoT is universal*. This view also sheds some light on the apparent pervasiveness of logic-like capacities across age-groups and species, and these constructions constitute special types of categories called toposes (topoi), and every topos has an interpretation in first-order logic.

## 1 Introduction

The Language of Thought (LoT) hypothesis supposes that (some) collections of mental states and processes constitute symbol systems having a *combinatorial syntax and semantics* that is akin to a language: LoT is a symbol system that represents complex entities by compositions of symbols representing the entities' constitutents so that syntactic relations among constituent symbols capture in a structurally consistent manner the semantic relations among corresponding constituent entities (Fodor, 1975, 2008). Moreover, for instance, the state of affairs *John loves Mary* is represented by a composition of symbols John, Mary and Loves for constituents *John* and *Mary*, and their *loves* relationship that are composed of a complex symbol Loves(John,Mary) representing *John loves Mary*. This situation is distinguished from a different, but related, state of affairs *Mary loves John* by the composite symbol Loves(Mary,John) that swaps the positions of John and Mary in accordance with the exchange of roles. The roles are encoded by the relative position of the filler symbols, which allows one to infer the lover of the relationship independently of the particular instance. Symbolic expressions assumedly correspond to physical states in a consistent way via a *physical instantiation mapping* (Fodor and Pylyshyn, 1988). Causal relations between these physical states are supposed to underwrite the inferential relations afforded by transformation of corresponding symbolic expressions—LoT is realized as a *physical symbol system* (Newell, 1980), analogous to the instantiation of a programming language with a computer, and situates within a *representational/computational theory of mind* (Wilson, 1999).

A motivation for LoT is to explain *systematicity* properties of thought: e.g., why having the ability to represent *John loves Mary* and infer that *John* is the lover in that relationship implies having the ability to represent *Mary loves John* and infer that *Mary* is the lover in this relationship (Fodor and Pylyshyn, 1988). As a simple illustration, LoT assumes (atomic) symbols for *John* and *Mary*, John and Mary, an operation that combines those symbols into strings of symbols, (John,Mary)↦John Mary, and an operation that transforms the string to recover the first symbol as the lover, as in John Mary↦John. Moreover, having representational and inferential abilities with regard to *John loves Mary* is supposed to imply having representational and inferential abilities with regard to *Mary loves John* because both involve the same processes. However, an explanation for systematicity requires more than simply positing a collection of (atomic) symbols, processes for constructing complex symbolic representations from those atoms, and processes for transforming those representations. LoT assumes that the construction processes are the “canonical” ones affording all possibly needed combinations (not just some of them) and that all possibly needed transformations are always in lock-step with those constructions. These assumptions have led to conclude that LoT lacks a complete explanation for systematicity in failing to explain why these assumptions should hold (Aizawa, 2003), i.e., LoT suffers from the same type of problem raised for connectionist (neural network) theory (Fodor and Pylyshyn, 1988). Others, however, contend that these assumptions *are* the core principles of LoT, not *ad hoc* assumptions (McLaughlin, 2009).

This contention has become more pressing with evidence of LoT-like properties in more diverse (non-linguistic) forms of cognition than previously realized (Quilty-Dunn et al., 2023)—LoTs appear seemingly everywhere in everyone and by extension some capacity for logic (Mandelbaum et al., 2022). However, accounting for the emergence of these properties is still a question for further study (Quilty-Dunn et al., 2023), which depends on explaining why/how symbolic representations connect to non-symbolic formats, given the usual distinction between (System 1) processes that are fast, non-symbolic, and developmentally early vs. (System 2) processes that are slow, symbolic, and developmentally late (Kahneman, 2011; Evans and Stanovich, 2013). Category theory (Awodey, 2010; Leinster, 2014) was invented to compare mathematical constructions (Eilenberg and Mac Lane, 1945), e.g., across algebra and topology, cf. symbolic and spatial representation. In this vein, we return to a category theory explanation for systematicity (Phillips and Wilson, 2010) to account for LoT-like properties and their relationships to non-symbolic systems. A categorical explanation says that systematicity follows from *universal construction*, i.e., a type of “best” solution for the given situation. For instance, systematicity for *John loves Mary* pertains to a universal construction called a *categorical product* on the set of atomic symbols, {John,Mary}, i.e., the set of *all* pairwise combinations of symbols together with two projection maps retrieving the first and second symbols. The purpose of the current study is to apply this approach to bridge the explanatory gap between symbolic and non-symbolic properties and, in doing so, begin to address questions about how LoTs (may) differ within and between species (Mandelbaum et al., 2022). An outline follows:

The perspective presented here is supposed to bridge this gap as a duality in a category theoretical sense: (perceptual) data are projected onto a base (conceptual) space in one direction, and in the opposite direction, these data are referenced by that space. Accordingly, perception is dual to conception. For a preview, these maps pertain to *fiber bundles* and *presheaves*, respectively, and their maps are related by *universal mapping properties* affording an explanation for why/how symbolic and non-symbolic formats are connected: as a best possible transformation between formats. An upshot of this approach is that each additional LoT-like property also pertains to a universal construction which constitutes a special type of category called a *topos*. Every topos has an interpretation in first-order logic, hence the apparent pervasiveness of a capacity for logic across age groups and species. A summary of these LoT-like properties is given next and a category theory account in the section that follows. Discussion of this view is given in the final section. This study provides a novel category theory perspective on the LoT hypothesis. Category theory may seem abstruse to many cognitive scientists, so the presentation style is informal rather than technical to facilitate some conceptual intuitions. More technical details are found in references and Supplementary material, which provides the background theory pertaining to formal conceptions of LoT and some further perspective. The central intuition driving this category theoretical perspective on LoT is that the relations between representational states, i.e., the *maps* (also called *morphisms* or *arrows*) of a *category*, have “shape”, and shape is given by topology, which affords the common ground for moving between (LoT-like) symbolic and non-symbolic representational formats.

### 1.1 Additional LoT-like properties

LoT is usually considered for System 2 (contra, System 1) cognition. However, other LoT-like properties are also apparent in non-linguistic domains, including perceptual, social, infant, and non-human cognition (Quilty-Dunn et al., 2023). Six additional properties were educed from an analysis of the literature with a particular focus on visual cognition in the context of *object files* (Kahneman et al., 1992) that supposedly store and update information on external objects over time. The following is a summary of the six properties (Quilty-Dunn et al., 2023, with the quoted text from there). Language-like examples are given for their familiarity with a more detailed connection to object files given in the next section.

1. *Discrete constituents*: representations have “distinct constituents corresponding to individuals and their separable features”. For example, the representation, Loves(John,Mary) for *John loves Mary* has distinct constituents John and Mary corresponding to individuals *John* and *Mary*.
2. *Role-filler independence*: “[LoT architectures] combine constituents in a way that maintains independence between syntactic roles and the constituents that fill them”: e.g., the constituents John and Mary represent the same individuals, i.e., *John* and *Mary* independently of their syntactic roles as subject or object in the loves relation.
3. *Predicate-argument structure*: “a predicate is applied to an argument to yield a truth-evaluable structure”, e.g., it may or may not be true that John loves Mary.
4. *Logical operators*: AND, OR, IF, and NOT.
5. *Inferential promiscuity*: “computational processes that transform representations with one logical form into representations with another logical form”. For example, if *John is tall and Mary is tall, John is tall* corresponds to transformation from tall(John) AND tall(Mary) to tall(John).
6. *Abstract conceptual content*: the capacity to “represent abstract categories without representing specific details”, e.g., representing a concept of chair as an abstraction of specific chair instances.

Parallel examples in visual cognition involve symbol-like representations for external objects and their visual features in ways that exhibit the six LoT-like properties. The authors argue that the LoT hypothesis is currently the “best game in town” by providing a broader account of cognitive behavior (Quilty-Dunn et al., 2023), which aligns with an earlier assessment from the classicist vs. connectionist debate over properties, such as systematicity (Aizawa, 2003).

Despite the explanatory successes and renewed interest in LoT, the question of how LoT is supposed to arise remains (Quilty-Dunn et al., 2023). Note that a probabilistic version of LoT (Goodman et al., 2015) does not necessarily help here, since the probabilistic inferences are over LoT-like representations. This situation would seem to favor deep learning approaches that eschew symbols and learn directly from data (see Kriegeskorte, 2015; Krizhevsky et al., 2017; Vaswani et al., 2017). However, there is an ongoing debate as to whether deep learning models are psychologically plausible in regard to the training which are needed for human-level performance and the specific types of predictions and errors made (Bowers et al., 2022). Our focus here is a categorical perspective on LoT (which may also help reconcile these views in the System 1/System 2 sense, already mentioned), not an attempt to settle this debate.

## 2 A category theory perspective

Our category theory perspective begins with a brief overview of basic concepts used to recast LoT in category theoretical terms. We start with the concept of *map*, which is also called *morphism* or *arrow* (Awodey, 2010; Leinster, 2014). A map is a “directed relation” between a pair of *objects*, written *f*:*A* → *B*, to indicate that map *f* goes from object *A* to object *B*, called the *domain* and *codomain* of *f*, respectively. The two senses of the word “object” used throughout the current study are distinguishable from the context. A pair of maps *f : A* → *B* and *g*:*B* → *C* (i.e., where the codomain of *f* is the domain of *g*) compose to form a map: *h* = *g* ◦ *f*:*A* → *C*, where ◦ denotes the composition operation. A collection of objects and a collection of maps together with a composition operation satisfying certain rules constitute a *category*, **C**. The archetypal example is the category of sets and functions, **Set**, where the composition operation is function composition. Categories may also be objects in a larger category. In this situation, the arrows in the larger category are called *functors*, *F*:**C** → **D**, which map objects to objects and morphisms to morphisms in a structurally coherent way: e.g., monotonic functions (order-cohering maps), *f*:*P* → *Q*, are functors between categories that are ordered sets, *a* ≤_*P*_*b* implies *f*(*a*) ≤_*Q*_*f*(*b*). Functors are also objects in categories whose maps are called *natural transformations*, $\eta :F\overset{.}{\to }G$, which transform the images of the domain functors, *F*(*A*) and *F*(*f*), into the images of the codomain functors, *G*(*A*) and *G*(*f*), in a coherent way, i.e., the result of a transformed map is the same as the transformed result of a map, *G*(*f*) ◦ η_*A*_ = η_*B*_ ◦ *F*(*f*). Deeper and broader expositions are found in many introductions to category theory (Awodey, 2010; Leinster, 2014), along with a conceptual introduction specifically for cognitive scientists (Phillips, 2022), scientists generally (Spivak, 2014), and a broader audience (Lawvere and Schanuel, 2009). Here, categories are regarded as collections of symbolic or non-symbolic states and functors and natural transformations as maps between them. Our categorical approach is presented in three parts by, first, introducing two principles guiding our perspective on LoT (Section 2.1), second, recasting the six LoT-like properties using these principles (Section 2.2) and, third, observing that these constructions constitute a special type of category, called a *topos*, which also pertain to object files (Section 2.3).

### 2.1 Universal mapping properties and principles

There are two category theory principles that guide our perspective on LoT: the *universal mapping principle* and the *duality principle*. The universal mapping principle (*UMℙ*) says that constructions are determined by a *universal mapping property* (UMP) that is given by a *unique-existence* condition. For example, the categorical product of sets *A* and *B* (introduced earlier) is a set *P* and two maps π_*A*_:*P* → *A* and π_*B*_:*P* → *B*, called *projections*, that together satisfy the following unique-existence condition: for every set *Z* and pair of maps *f*:*Z* → *A* and *g*:*Z* → *B*, there *exists* a *unique* map *u*:*Z* → *P* such that (*f, g*) = (π_*A*_, π_*B*_) ◦ *u*. The product—pair (*P*, π), where π = (π_*A*_, π_*B*_)—has a universal property in that all maps into *A* and *B* factors through π. This construction is optimal in the sense that the unique-existence condition is only satisfied with sets that have at least as many elements as the number of *A*-elements *multiplied* by the number of *B*-elements (existence) and at most that many elements (uniqueness). There is generally more than one universal construction for the given situation (here, the maps into *A* and *B*), but all such universal constructions are *unique up to a unique isomorphism*, meaning that there is one and only one way to go between two such constructions. Every construction given by a UMP acts like a global minimum, i.e., all constructions point to the universal construction as a structural analog (abstraction) of gradient descent—there are no “local minima” in this situation. *UMℙ* says that universal constructions are obtained by following this “gradient”. In this way, the category theory approach (Phillips and Wilson, 2010, 2016) is supposed to address the basic explanatory criterion for systematicity—i.e., to explain why systematicity necessarily follows from the core principles of the theory, not just how systematicity may be consistent with that theory (Fodor and Pylyshyn, 1988; Aizawa, 2003)—essentially, as a universal construction, *All roads lead to Rome!* This principle is supposed as a driver of cognitive constructions (Phillips, 2021).

The duality principle pertains to the directionality of maps. Formally, for every category **C**, there is an *opposite category* **C**^**op**^ that has the objects and the “reversed” arrows of **C**, i.e., an arrow *f*:*A* → *B* in **C** is an arrow *f*^**op**^:*B* → *A* in **C**^**op**^. A construction in **C** has a dual construction in **C**^**op**^ (NB. **C**^**op**^**op** = **C**). This principle also applies to universal constructions. For instance, a product in **C** has a dual universal construction called the *coproduct*, which is the product in **C**^**op**^. Coproducts are universal constructions so follow the same pattern for products with the arrows reversed. To wit, the coproduct of sets *A* and *B* is a set *Q* together with two maps (injections, ι_*A*_:*A* → *Q* and ι_*B*_:*B* → *Q*) infusing *A* and *B* into *Q* such that for any set *Z* and maps *f*:*A* → *Z* and *g*:*B* → *Z* the pair (*f, g*) factors uniquely through the injections: i.e., there exists a unique map *u*:*Q* → *Z* such that (*f, g*) = *u* ◦ (ι_*A*_, ι_*B*_). This construction is also optimal in that the unique-existence condition is only satisfied with sets that have at least as many elements as the number of *A*-elements *plus* the number of *B*-elements (existence) and at most that many elements (uniqueness). A category and its opposite are related to a functor, *I*^**op**^:**C** → **C**^**op**^. More generally, a functor of the form *F*:**C** → **D**^**op**^ (equivalently, *F*:**C**^**op**^ → **D**) is called *contravariant* from **C** to **D** (a functor of the form *F*:**C** → **D** is called *covariant* from **C** to **D**). A pair of opposing functors (possibly contravariant), *F*:**C**⇆**D**:*G*, that are related by a certain type of universal construction is called an *adjoint situation*, i.e., the best possible recovery of the objects *C* and maps *f* in **C** from their images, *F*(*C*) and *F*(*f*) in **D**, by the opposing functor *G* as the objects *GF*(*C*) and maps *GF*(*f*) in **C**; equivalently, the best possible recovery of the objects *D* and maps *g* in **D** from their images, *G*(*D*) and *G*(*g*) in **C**, by the opposing functor *F* as the objects *FG*(*D*) and maps *FG*(*g*) in **D**. Adjoint situations are also regarded as a form of duality. In this way, the universality and duality principles are intimately related.

These two principles guide our categorical perspective on thought as mappings between percepts and concepts representing entities. We start with a basic, dual-view of a function between sets *f*:*A* → *B* as an *A*-labeling of the elements in *B* or the *B*-properties of the elements in *A* (Lawvere and Schanuel, 2009). In a deck of cards, for example, each card has the property of *suit* (i.e., *club, spade, diamond*, and *heart*) and *rank* (i.e., *two, three*,..., *queen, king, ace*). Accordingly, there are maps from the deck listing these properties, i.e., *suit*:*D* → *S* and *rank*:*D* → *R*. We may also think of the deck as a set of (perceptual) instances for the concepts of suit and rank. Moreover, a map in one direction (percepts to concepts) is a conceptualization of percepts and a map in the opposite direction is a perceptual instantiation of concepts. In general, a function in **Set** does not have an opposite as a function in **Set**^**op**^, unless that function has an inverse. The best one can do is to recover the *preimage* from every element *b* in *B*, i.e., the set of elements in *A* whose image under *f* is *b*. For instance, suppose a set of colored shapes, *CS* = {♠, ♣, ♢, ♡}, a set of colors, *C* = {*b, r*}, and a set of shapes, *S* = {*s, c, d, h*}. The color function produces a color image for each item, e.g., *f*_*c*_:♠↦*b*, and the preimage function for *f*_*c*_ recovers the subset of colored shapes with the given color, e.g., $f_{c}^{-1}\left[C\right]:b\mapsto \left\{♠,♣\right\}$. Similarly, for shape, *f*_*s*_:♡↦*h* and $f_{s}^{-1}\left[S\right]:h\mapsto \left\{♡\right\}$. These two perspectives illustrate a simple case of a more general duality (adjoint situation) that exists between fiber bundles and presheaves, where the sets have the structure of a topological space. There are also maps between bundles and maps between presheaves that are determined by universal properties (Mac Lane and Moerdijk, 1992; Awodey, 2010), which can be interpreted as changes in perception and conception. Simple examples of these situations are used for our categorical view of LoT-like properties.

### 2.2 A LoT like a category

Thought is supposed to have (at least) six additional LoT-like properties. Quilty-Dunn et al. (2023) consider properties in visuospatial formats pertaining to object files (Kahneman et al., 1992). An object file is characterized as a representation that “(i) sustains reference to an external object over time, and (ii) stores and updates information concerning the properties of that object” (Green and Quilty-Dunn, 2021). Object-tracking and visual memory effects, e.g., forgetting/remembering of a color feature independently of an orientation feature or tracking an object despite a feature change is observed to support a propositional/compositional format over formats that are iconic/wholistic (Green and Quilty-Dunn, 2021; Quilty-Dunn et al., 2023). Other domains include reasoning and inference over visually presented information. The purpose here is to show how these six LoT-like properties pertain to the universality and duality principles with examples of case situations from Quilty-Dunn et al. (2023).

#### 2.2.1 Discrete constituents

The discrete constituents property, in the context of object files, pertains to visual features such as color and shape that are represented independently of each other as implied by, say, a recall task where accuracy in recalling color does not change with shape and accuracy in recalling shape does not change with color. LoT captures the discreteness property by positing symbols for the constituent features and a process that combines symbols to construct complex representations, e.g., *rs* for a *red square* and *rt* for a *red triangle*. The symbol for *red* does not change with the juxtapositioning of shape symbols capturing discreteness. The order of the symbols does not matter for this property.

From a categorical (topological) perspective, discreteness is a universal (mapping) property. A set *X* can be given a spatial (topological) structure by imposing a *topology* *T* consisting of subsets of *X*, called *open sets*, that specify the relative proximity of elements in *X*. The inclusions of open sets imply an order. In this way, an order is imposed on the elements of a set by specifying a corresponding topology. Every set has two extreme topologies: the *discrete topology* where every subset of *X* is an open set and the *indiscrete topology* where the only open sets are the empty set and *X*. In the discrete case, every element *x*∈*X* belongs to an open set with just that element, {*x*}, and corresponds to the discrete constitutent property (every symbol is “equiproximal” to every other symbol). Discreteness in this topological sense is a universal construction. A space *T* is *finer* than a space *T*′ if every open set of *T*′ is an open set of *T*; dually, *T* is *coarser* than *T*′ if every open set of *T*′ is an open set of *T*. The discrete topology is the finest space on *X*, since every subset of *X* is an open set. The indiscrete topology is the coarsest space, since the only open sets are the empty set and *X*, which are required for any topology. Moreover, in the category of topologies on *X* ordered by inclusion of open sets, the indiscrete and discrete spaces are universal constructions called the *initial* and *terminal* objects, respectively: an object is initial (terminal), denoted 0 (1) if for every object *Z* in the category there exists a unique map *u*:0 → *Z* (*u*:*Z* → 1). Hence, discreteness follows from a universal construction.

The importance of this topological view is in placing the notion of symbolic (discreteness) on common ground with a notion of non-symbolic (non-discreteness). A non-discrete space is an indiscrete space or a space that is not a discrete space. A *continuous function* between topological spaces, *f*:*X* → *Y*, preserves proximity at possibly courser levels a granularity, meaning that for every open set *U* of *Y*, the preimage of *U* is an open set of *X*. The collection of topological spaces on *X* and their continuous functions constitute a category, which affords the common ground for moving between symbolic and non-symbolic systems.

#### 2.2.2 Role-filler independence

Evidence for role-filler independence arises in a way that is similar to discrete constitutents in that the representations of roles are not affected by particular fillers and the representations of fillers are not affected by particular roles. The canonical example is the representations of *John loves Mary* and *Mary loves John* where representing the role of lover is the same regardless of being filled by the symbol for *John* or *Mary* (e.g., as always being encoded as the first position) and the representation for *John* is the same regardless of the role (as in always being the same symbol). In the context of object files, role-filler independence appears in object tracking tasks where the identity of the object (role) remains unchanged despite changes in visual features such as color (filler)—role independence from filler. Conversely, attributes can maintain their integrity despite swapping attributed objects—filler independence from role (Quilty-Dunn et al., 2023). In this situation, roles can also be (explicitly) represented as symbols that are composed of symbols for fillers to indicate their role.

A corresponding categorical view is to treat the roles and fillers in terms of *presheaves*, i.e., set-valued (contravariant) functors on topological spaces, 𝓕:*X*^**op**^ → **Set**. A presheaf maps open sets and inclusions, interpreted as roles and role relations, to sets and restrictions maps, interpreted as fillers and filler relations. For instance, the part-whole relations among the symbols ⊠, ⊞, × , + are given by a presheaf that sends the inclusion {*}⊆{*, ⋆} to the *restriction map* ⊠↦ × , ⊞↦+. In this way, a presheaf has spatial and non-spatial properties (more complex relationships beyond part-whole relations are also given by presheaves on other topological structures, such as graphs, or complexes). A *sheaf* is a universal presheaf, which has the special property that the attached data are recoverable from just the data attached to the points of the underlying space, called the *stalks* of the sheaf. The dual view is the category of fiber bundles on that space: presheaves map roles to fillers and bundles map fillers to roles. A perspicuous way to think about presheaves is as relational database tables, where table headings correspond to the points of the space and table rows correspond to the image of the presheaf (Abramsky and Brandenburger, 2011; Abramsky, 2013). For instance, the loves relation can be regarded as a presheaf on the discrete topological space for the two-point set {s,o}, where s represents the subject and o represents the object of the relation, that is mapped to a set of pairs including (John,Mary) representing the instance *John loves Mary*. Two types of maps of presheaves are presheaf morphisms which are maps between the data attached to the same space (table rows) and maps between possibly different spaces on which the data are attached (table headings). Suppose, for instance, *John loves Sue* instead of *Mary*. This change is conveyed by the mapping, (John,Mary)↦(John,Sue). The passive form, *Mary is loved by John* is obtained from a continuous function mapping subject to object and object to subject, s↦o,o↦s, inducing a functor that swaps the corresponding columns creating a new table for the passive form. Accordingly, roles and fillers of a relation are independently represented by presheaves.

This situation also applies to the type of role-filler independence that is claimed for object files (Quilty-Dunn et al., 2023). The roles for object files are points of a (discrete) topological space and their attributes are the attached data. Change in a color feature, say, from *red* to *green* for a tracked object corresponds to a presheaf morphism on a one-point space: a mapping between corresponding one-tuples, e.g., (r)↦(g). The “swapping” of color features in multiple object tracking scenarios (Quilty-Dunn et al., 2023) corresponds to the functor induced by the continuous function that exchanges the role of each object as points (locations) in a topological space, l_1_↦l_2_,l_2_↦l_1_. This situation generalizes to other scenarios, e.g., the continuous function on a three-point space, l_1_↦l_2_,l_2_↦l_1_,l_3_↦l_3_ leaves the attributes attached to the third role unchanged. Moreover, object files as presheaves capture role-filler independence.

Structured (part-whole) relations also appear among objects in a complex visual scene that are akin to grammatical structure, which provides further support for a LoT in visual cognition (Quilty-Dunn et al., 2023). Hierarchical structures are orders, which have corresponding topologies. Hence, the LoT-like relational structure of visual scenes is also captured by presheaves on such topologies.

#### 2.2.3 Predicate-argument structure

Quilty-Dunn et al. (2023) also argue that object files have a predicate-argument structure akin to language, as in their examples “That spherical object is red” or “That red object is spherical”. The object is predicated by a color in the first example and by a shape in the second example. A map *f*:*A* → *B* can be regarded as listing the *B*-properties of *A*-objects (Lawvere and Schanuel, 2009). Every continuous function *f*:*Y* → *X* induces a *sheaf of sections* on *X*, 𝓕:*X*^**op**^ → **Set**, i.e., for each open set *U* of *X*, the set of functions 𝓕(*U*) = {*s*:*U* → *Y*|*f* ◦ *s* = 1_*U*_}, called the *sections* (right-inverses) of *f* restricted to the open set *U*. Moreover, suppose a set of colored shapes, *CS*⊆*C*×*S* where *C* and *S* are the sets of colors and shapes with the discrete topologies. The (trivial) fiber bundle on *C* consists of the projection π_*c*_:*C*×*S* → *C* which induces the sheaf of sections on *C*, i.e., the dual map, 𝓕:*C*^**op**^ → **Set** sending the color red, for instance, to the set of red objects. Similarly, the projection π_*s*_:*C*×*S* → *S* induces the sheaf of section on *S*. Thus, the duality between bundles and presheaves captures predicate-argument structure.

In the sense of (classical) logic, predicate-argument structure pertains to propositions that either true or false. For relations as subsets of Cartesian products, evaluation is given by the relation's *characteristic function*: for a (binary) relation *R*⊆*A*×*B*, the characteristic function is a function χ_*R*_:*A*×*B* → 2, where 2 = {⊥, ⊤} is the set of truth values, sending pair (*a, b*) to ⊤ (true) whenever *a* is *R*-related to *b*, i.e., (*a, b*)∈*R*, otherwise ⊥ (false). There is a one-to-one correspondence between relations and their characteristic functions, which is given by the following “square” of arrows (see Goldblatt, 2006):


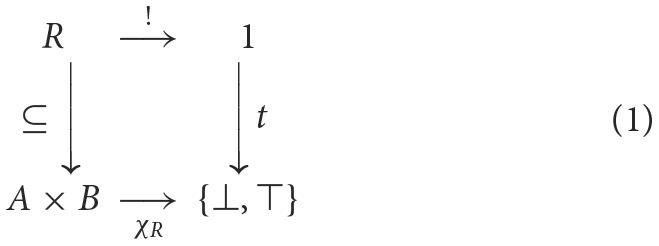


where *t* picks out ⊤ (true). This unique correspondence follows from another instance of a universal construction involving the terminal object, 1 = {*}, and the *pullback* of the arrow *t* along χ_*R*_ yielding *R*. Pullbacks are given by a universal mapping property that is closely related to products. For sets, the pullback of a function *f*:*A* → *C* along a function *g*:*B* → *C* (equivalently, *g* along *f*) is a set *A*×_*C*_*B* = {(*a, b*)|*f*(*a*) = *g*(*b*)} and its two projections, which acts like a product constrained by the maps into *C* (if *C* is the terminal object, implying no constraints, then the pullback is just a product).

More generally, for a category **C** with a terminal object, an object Ω in **C** is called a *subobject classifier* for **C** if there exists an arrow τ:1 → Ω such that for each *monomorphism* (cf. injection) ι:*U* → *X* there exists a unique arrow χ_ι_:*X* → Ω such that the following diagram is a pullback (see Goldblatt, 2006):


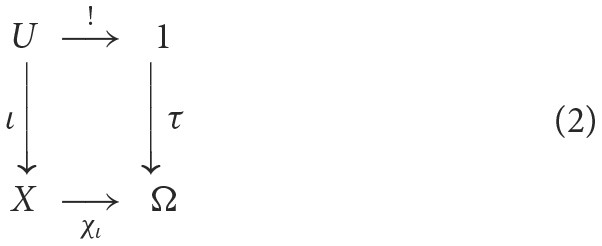


where χ_ι_ is called the *classifying morphism*, τ is called the *truth arrow*, and *U* is called a *subobject* of *X*. This abstraction from subset to subobject affords a richer notion of truth. In the category of presheaves on *X*, for instance, the subobject classifier is interpreted as assigning truth to just that part of the data on the open sets for which the predicate holds. If the predicate holds nowhere or everywhere on the space, the empty set or *X* is returned, respectively, corresponding to false or true in the usual sense.

#### 2.2.4 Logical operators

Logical operators AND, OR, IF, and NOT have corresponding abstractions in category theory. We have already observed that the two-element set corresponds to the set of Boolean truth values, 2 = {⊥, ⊤}. This set is constructed from the coproduct of singleton sets, 1 = {*} (terminal objects), i.e., 2 = 1+1, where + denotes the coproduct. The four logical operators correspond to maps between these sets: e.g., AND (∧) is the map ∧:2 × 2 → 2;(⊥, ⊥)↦⊥, (⊥, ⊤)↦⊥, (⊤, ⊥)↦⊥, (⊤, ⊤)↦⊤; similarly, for OR and IF, NOT is the map ¬:2 → 2;⊥↦⊤, ⊤↦⊥. These operators in coordination with the subobject classifier afford logical operations over relations. The complement of a relation *R*⊆*A*×*B*, denoted $\bar{R}$, is given by composing the subobject classifier χ_*R*_ with the map for NOT, i.e., the subobject classifier $\chi_{\bar{R}}=\neg •\chi_{R}$.

The two-cups task is supposed to demonstrate a capacity for logic, i.e., if an object is NOT under one cup, it must be under the alternative cup (Quilty-Dunn et al., 2023). Suppose CONTAINS is the predicate on the set of cups yielding true if the object is under the cup. Composing with the NOT map yields the subobject classifier identifying the alternative cup as containing the object.

#### 2.2.5 Inferential promiscuity

Inferential promiscuity in the visual context is supposed when inferences transcend changes in visual features. LoTs have the inferential promiscuity property by virtue of computational processes that transform representations with logical form: e.g., *P*∧*Q*⇒*P*, or *P*⇒*P*∨*Q*. The categorical analogs of conjunction and disjunction are the categorical product and coproduct. In a category with products and coproducts, **C**, the product functor sends pairs of objects (and maps) to their products, Π:(*A, B*)↦*A*×*B*. The implication *P*∧*Q*⇒*P* corresponds to the natural transformation from the product functor to the functor picking out the first object, $\overset{´}{\Pi }:\;\left(A,B\right)\mapsto A$, i.e., the natural transformation $\pi :\Pi \overset{.}{\to }\overset{´}{\Pi }$ whose maps are the natural projections π_*A*_:*A*×*B*↦*A*. The analogous situation applies to disjunction, which corresponds to the natural injection, ι_*A*_:*A*↦*A*+*B*. Moreover, functors act as representations, hence natural transformations as representation transformations that range over the objects *A* in **C**, such as *P* ranges over propositions.

#### 2.2.6 Abstract conceptual content

Object-specific preview benefit, i.e., a reaction-time benefit for test items related to previously viewed stimuli, particularly across modalities such as observing an image of an apple for basic category APPLE and testing with the word “apple” suggests that object-files store abstract conceptual content along with specific instances of that concept (Quilty-Dunn et al., 2023). LoT accommodates this situation by assuming that perceptual representations of the instances are linked to a symbolic representation of the concept.

We have already observed a categorical version of abstract conceptual content in the deck of cards, for instance, the map from cards to suits, *suit*:*C* → *S*, which assigns specific cards to their suit, e.g., *suit*:2♡↦♡. This function (projection) constitutes a fiber bundle and induces a sheaf of sections that sends each suit to the set of cards with that suit, as in {♡}↦{2♡, …, 10♡, J♡, …, A♡}. Naturally, this situation also extends to individual cards that are given by different decks, i.e., where cards with different faces, say for the *two-of-hearts*, indicate the same card, 2♡. In this way, perception is dual to conception. The map from percept to concept (fiber bundle) acts as the conceptual interpretation of the stimuli, and the map from concept to percept (presheaf) acts as the best possible recovery of the stimuli after the percept is no longer available due to an occlusion event.

### 2.3 A topos of thought (object files)

The universal and dual constructions pertaining to the six LoT-like properties constitute a special type of category called a *topos* (Mac Lane and Moerdijk, 1992; Lawvere and Rosebrugh, 2003; Goldblatt, 2006). A topos is a category that has all *(finite) limits* and *exponential objects*. We have already observed examples of limits in the form of terminal objects, products, and pullbacks and their dual constructions, called *(finite) colimits*, in the form of initial objects and coproducts. (The dual of pullback is *pushout*.) An exponential object in **Set** consists of a collection of functions from a set *A* to a set *B*, denoted *B*^*A*^, called a *function space*—the number of functions in *B*^*A*^ is |*B*|^|*A*|^, where |*A*| and |*B*| are the number of elements in *A* and *B*, respectively. Note the two special cases where *A* and *B* are the initial and terminal objects, i.e., the empty set and the singleton set, respectively—the number of functions in *B*^0^ and 1^*A*^ is |*B*|^0^ = 1^|*A*|^ = 1, as required by the definitions for initial and terminal [exponential objects pertain to another type of universal construction, which have applications in computer science as the *curry-uncurry* transform: e.g., addition as an operator whereby *a*+*b* = (+_*a*_)*b*]. Having finite limits and an exponential object implies having finite colimits and a subobject classifier. These conditions define an *elementary topos*, which are easier to state, though more general, than the conditions for the original *Grothendieck topos* (differences between these concepts are not explored here). Object files were regarded in terms of fiber bundles and presheaves, in the previous section. Categories of presheaves on a space *X* and categories of fiber bundles over *X* are topoi, which evokes another slogan, *LoT is a topos*.

A category/topos theory approach arguably affords the most concise, systematic, and formally precise framework for studying the structure of thought. The basic ingredients of a topos are constructions derived from universal mapping properties. All (finite) limits are constructed from just two special cases, i.e., pullbacks and terminal objects—equivalently, products and another type of limit called *equalizer* (see Leinster, 2014). Moreover, limits are constructed from adjoint functors which are themselves obtained from *Kan extensions* (Mac Lane, 1998). For instance, the product functor Π:**C**^2^ → **C** is obtained from a *Kan extensions* of the identity functor 1_**C**_ along the diagonal functor Δ:**C** → **C**^2^, as indicated by the following “triangle” of maps:


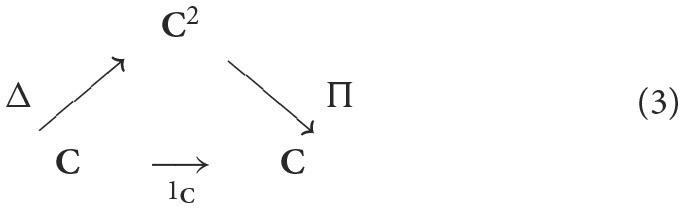


This situation is akin to an autoassociative neural network model that attempts to recover the input from its hidden unit representations by adjusting parameters to minimize the distance between the target given by the identity map and the network response. The best that one can do in this categorical situation is a natural transformation from the target given by the identity functor to the system response given by the composition *G* ◦ *F*, i.e., $\eta :1_{\text{C}}\overset{.}{\to }G•F$; dually, $\epsilon :F•G\overset{.}{\to }1_{\text{D}}$. A Kan extension in this situation may be regarded as the best that a (cognitive) system can do to recover what was lost by taking an action as a (left/right adjoint) functor *F*:**C** → **D**, i.e., the right/left adjoint functor *G*:**D** → **C**, thus absorbing the universality and duality principles as a single *universal-duality (adjointness) principle*.

This adjointness principle affords a basis for explaining why/how symbolic representations connect to other, non-symbolic representational formats. Recall that categories of topological spaces and continuous functions provide common ground for connecting symbolic and non-symbolic representations as discrete and non-discrete spaces, respectively. For instance, there is a continuous function from the two-element set {*, ⋆} with the discrete topology to the same set with the indiscrete topology, i.e., the map sending each element to itself, thus realizing a transition between a symbolic to a non-symbolic format. This difference is analogous to the difference between a *multi-slot* vs. *single-slot* view of object files (Green and Quilty-Dunn, 2021)—each point (open set) in the discrete space corresponds to a different object role vs. a single object role (non-empty open set) in the indiscrete case by their topologies. In the discrete case, each point belongs to a distinct open set, {*} and {⋆}. For the indiscrete case, the only non-empty open set contains both points, {*, ⋆}. For the corresponding categories of presheaves, the discrete space affords compositionality, whereas the indiscrete space does not. For example, the deck of cards represented as a presheaf on the set representing suit and rank, {*s, r*}, with the discrete topology affords recovery of the suit and rank of each card: for instance, the inclusion {*s*}⊆{*s, r*} is sent to the restriction map that consists of the mapping 2♡↦♡. No such access to constituents is afforded with the indiscrete topology because the indiscrete topology does not have any non-trivial inclusion maps.

Some theoretical and empirical implications of this difference in topology pertain to the respective presheaf morphisms, as illustrated by a simple example. Suppose the string “CAT” is associated with the string “DOG”. What should the strings “ACT” and “TAC” be associated with? If the strings are interpreted as words and the mapping as a free association, one might respond with “NOW” and “TIC”, respectively, by ignoring associations between constituent letters. This situation corresponds to a map between presheaves on an indiscrete space. However, if the strings are interpreted as letter sequences and the association as a mapping of letters, one might respond with “ODG” and “GOD”, respectively, using the three constituent letter maps for the map from “CAT” to “DOG”. This situation corresponds to a map between presheaves on a discrete space. Thus, the empirical implications pertain to responses on novel strings: the discrete case affords generalization—correct prediction of target responses for novel strings; the indiscrete case does not. These two situations were observed experimentally when subjects trained on letter-pair to color-shape associations exhibited both forms, depending on the difficulty of the task, i.e., number of associations to be learned (Phillips et al., 2016). Moreover, the relationship between these two cases is given by an adjoint situation (Phillips, 2018). An analogous situation may be used to reconcile multi-slot and single-slot views of object files, where visual features spontaneously *do* vs. *do not* break apart.

## 3 Discussion

What is gained by rendering LoT-like properties in terms of category/topos theory constructions? The idea of treating relations between representational states as arrows affords two gains, which is discussed here. First, arrows have shape (in addition to direction) that is given by topology in the context of sheaves and bundles, providing the common ground needed to connect symbolic and non-symbolic formats via continuous functions. This idea that the (causal) relations between representational states are determined by the “shape” of those states which is familiar to the classical view as syntactic form (Fodor and Pylyshyn, 1988). What category theory adds is the way that certain shapes (constructions) are universal—satisfy universal mapping properties—and that every universal construction follows from the universal mapping principle: “gradient” descent (ascent) to the corresponding terminal (initial) object (Phillips and Wilson, 2016; Phillips, 2021). In this way, category theory affords an extra level of explanation for why such LoT-like properties arise, beyond the classical account of stipulating symbolic representations as needed (see Section 1), and how such constructions connect to other formats, beyond just assuming that they do.

Second, category/topos theory provides a systematic treatment of LoTs in terms of a small number of conditions (limits and exponential objects) and principles (universality and duality). We already noted that limits and exponential objects are obtained from universal properties and that all finite limits arise from just two types, terminals and pullbacks. The conciseness goes even further—all limits are universal with respect to just one type of functor, the diagonal functor Δ:**C** → **C**^*J*^ where *J* is the “shape” of the functor into **C**, i.e., a category, *J*, typically consisting of a small number of objects and arrows, whose identities are unimportant, used to pick out objects and arrows in the target category, **C**. A terminal is a limit with respect to the empty shape and a pullback with respect to a shape that has three distinct objects and two distinct arrows, i.e., · → ·←·. Dually, colimits derive from just two types, initial objects and pushouts. The shape of an initial object is also the empty category, 0^**op**^ = 0. The shape category for pushouts is opposite the one for pullbacks, i.e., ·←· → ·. The diversity of limits and colimits stems from the variety of categories, **C**, to which the diagonal functor is applied. Not all categories have all types of limits, but all limits derive from the same universality principle (𝕌𝕄ℙ), i.e., the principle of construction from a universal mapping property (UMP). Moreover, in each case, the limit/colimit functor is right/left adjoint to the diagonal functor, thus combining the universality and duality principles into a single adjointness principle, whence the slogans, *Adjoints are everywhere* and *All concepts are Kan extensions* (Mac Lane, 1998). For the purpose of assessing the differences in LoTs within and across species (Mandelbaum et al., 2022), one needs a systematic organizational framework. As a formal *framework* in this regard, category/topos theory is arguably the “best game in town” for concision and formal precision. Topos theory brings together algebra, topology/geometry, and logic (see Leinster, 2011), cf. language, perception, and reasoning, which affords an analogous panoramic view for cognitive science: an integrative view of LoT (Fodor, 1975) as an algebra of thought, perception as a geometry of thought (Gärdenfors, 2000), and reasoning as mental models of thought (Johnson-Laird, 2008).

There are, however, important caveats to this categorical view. In particular, capacities for inference are not the same across age-groups and species. For instance, older children but not younger children around the age of 5 years old have a capacity for transitive inference (Halford, 1984; Andrews and Halford, 1998): if *aRb* and *bRc* then *aRc*. How should these differences be treated within this category/topos theory approach? One may appeal to cognitive capacity limitations, such as the number of items that can be concurrently held in working memory (Cowan, 2001) or the number of variables that can be concurrently processed (Halford et al., 1998). Presheaves as relational tables have a “two dimensional” structure in terms of the number of columns and the number of rows. The number of columns can be interpreted as relational arity: a one-column table is a unary relation and a two-column table is a binary relation. *Relational complexity theory* says that differences in inferential ability correspond to differences in the capacity to process relational information (Halford et al., 1998, 2014), which has also been considered in terms of categorical products/pullbacks (Phillips et al., 2009), which underlie sheaves (Mac Lane and Moerdijk, 1992). The number of rows can be interpreted as the number of items that can be stored or referenced. These two types of cognitive capacities (Halford et al., 2007) are potentially relatable via sheaf theory.

Another caveat is that not all aspects of a construction derive from universal properties. For instance, the universal property in an adjoint situation pertains to the left/right adjoint functor for the given functor, *F*, if such an adjoint exists. The given functor, *F*, itself is not necessarily obtained from some universal properties (if *F* has a left/right adjoint, *G*, *F* may be dually viewed as a universal construction given *G*). In these situations, a pair of adjoint functors act like hypothesis generation and test, i.e., given a hypothesis as the functor *F*, test this hypothesis for a universal property as the left or right adjoint *G* to *F*. There may be more than one adjoint situation between a pair of categories of which only some are relevant to the task at hand. In this case, the category/topos theory approach needs to be augmented with a measure of relevance or fitness akin to a neural network model. Incorporating category/sheaf theoretic constructions into such models is another possibility (Clark et al., 2008; Hansen and Gebhart, 2020; Barbero et al., 2022; Bradley et al., 2022). Cognitive systems are supposed to be resource-dependent and goal-driven. A categorical theory of resources (see Coecke et al., 2016) may also afford an explanation for why a universal property, hence systematicity, fails to emerge. A LoT is after all a physical symbol system (as mentioned earlier) embedded in some environment. If the environment provides insufficient information to establish the needed construction, the universal construction may be suboptimal with regard to the construction that would have been obtained had more information been made available, i.e., in a larger context. In this case, the universal construction is from a functor interpreted as the available resources to some object interpreted as the task goal (see Supplementary material, remarks 132).

Viewing LoT as a topos leads to other properties beyond the six canvassed in the previous section. The archetypal topos is the category of sets and functions, **Set**, whose classifying object is the set of Boolean values (true and false). Thus, **Set** acts like ordinary classical logic. A category of presheaves on a space *X* is also a topos but with a different classifying object consisting of the open sets of *X* which affords the graduated notion of truth mentioned earlier (NB. The classifying object is not a “free parameter” but a universal mapping property of the particular category/context that one is working in). The *law of excluded middle*, i.e., ¬¬*A* = *A*, characterizing classical logic, need not hold for some topoi. For instance, the category of directed graphs is a topos whereby ¬¬*A*≠*A*, but ¬¬¬*A* = ¬*A* (Lawvere and Schanuel, 2009). What these additional properties mean for a language of thought has yet to be worked out. However, a general implication of this graded notion of truth is that response errors should align with the open sets (roles), e.g., correctly recalling object color but not shape, which accords with the view that accounting for the types of errors that humans make is more important than simply modeling overall response accuracy, at least in vision (Bowers et al., 2022). A topos theory approach is also apt for a formal theory of goals that are organized into subgoals given that the concept of subobject is a generalization of the concept of subset, hence dependency order. To continue the analogy, functors are to *subfunctors* (cf. subsets) as goals are to subgoals, or partial solutions to a problem (see Supplementary material, remarks 132). Exploring the implications of these formal abstractions are topics for future studies.

The claim that LoT is universal is to be understood in the context of a particular aspect of cognition, not that all of cognition is necessarily LoT-like. LoT is not the only game in town (Quilty-Dunn et al., 2023) and universality is not absolute with respect to cognition *in toto*—universal constructions are local with respect to some category or relative with respect to some functor. As already discussed, systematic failures point to other aspects of cognition that are better accounted for by incorporating other types of theories, such as embodied cognition (Shapiro and Spaulding, 2024), that realize the requisite universal properties. Other approaches such as dynamical systems (Port and van Gelder, 1998) and Bayesian probability (Griffiths et al., 2008) also have categorical connections (Lawvere and Rosebrugh, 2003; Culbertson and Sturtz, 2014; St. Clere Smithe, 2023). Where category theory stands apart from other theoretical approaches is the way that such alternatives are incorporated into a universal way, vis-a-vis, adjointness. Indeed, topos theory is the quintessential example for mediation of opposites (Lawvere, 1989, 1992—see Rosiak, 2022, for further philosophical discussion). Category theory was developed as a type of meta-mathematics (Eilenberg and Mac Lane, 1945), and cognitive science was founded as an interdisciplinary science of mind (Miller, 2003). The pertinent parallel here is that category/topos theoretical approaches are the natural approach to explain why/how these different aspects live together. LoT may well have a good game, but category theory is the better sport.

## Author contributions

SP: Conceptualization, Formal analysis, Writing – original draft.
