# Supplementary material for: A category theory perspective on the Language of Thought: LoT is universal
Source: Front Psychol. 2024 Apr 30;15:1361580. doi: 10.3389/fpsyg.2024.1361580 (PMC11091282; doi:10.3389/fpsyg.2024.1361580)
Supplement: Supplementary file 1 [file Data_Sheet_1.pdf]

Supplementary Material

1 PREAMBLE

This material provides the formal background and organizational framework for the main text. The view presented here highlights the primacy of space (topology) in a sense that befits our categorical treatment of LoT. Although this material is not meant as an introduction to category theory, the presentation style is also intended to support the claim that from just a small number of elementary concepts and principles category theory affords a concise and systematic way to organize LoTs. There are other ways to present category theory concepts. Indeed, for the purpose of parsimony, one can treat categories in an arrows-only way and dispense with objects and composition operations altogether, since every object is associated with one and only one identity arrow (Mac Lane, 1998) and the composition operation is itself an arrow, i.e. a map sending certain pairs of arrows to arrows. However, an arrows-only view obscures the important and distinctive roles played by objects (Lawvere, 1991) and composition operations. The basic theory (section 2) affords a formal framework for positing LoTs and object files (section 3).

2 BASIC THEORY

The formal concepts provided here are found in many textbooks on category theory and related topics from introductory (Awodey, 2010; Lawvere and Schanuel, 2009; Leinster, 2014; Spivak, 2014) to more advanced levels (Goldblatt, 2006; Hartshorne, 2013; Mac Lane and Moerdijk, 1992; Mac Lane, 1998), and connections between presheaves/sheaves and relational database theory were previously established (Abramsky, 2013; Abramsky and Brandenburger, 2011). However, these concepts may not be familiar to many cognitive scientists, so are abstracted from the more familiar concepts of sets, functions and relations (section 2.1) in the form of categories, functors, natural transformations and universal constructions of various kinds (section 2.2). Moreover, despite the "many different empirical realizations [of mathematical structure]" (Mac Lane, 1992) and their categorical connections (Mac Lane, 1997) the relevance to cognition and the principles that motivate them may seem obscure. So, basic theory is presented in a way that highlights the universal-duality principle as a formal relationship between percepts and concepts in the form of presheaves (section 2.3) and their dual constructions, bundles (section 2.4), which constitute topoi (section 2.5). Context also plays a crucial role: every construction is given with respect to a category of some kind. For instance, *limits* are a general class of universal constructions *internal* to a category in one context or a special case of universal constructions *external (relative)* to another category that generalize to other kinds of universal constructions, such as *universal morphisms*. The presentation here also attempts to draw out such changes in context, as they play an important role in cognition.

2.1 Sets

Definition 1 (power set)**.** The *power set* of a set X, denoted $\mathcal{P}(X)$, is the set of all subsets of X, i.e., $\mathcal{P}(X) = \{A | A \subseteq X\}$.

Examples 2 (power set)**.** The following examples are power sets.

1. $\mathcal{P}(\emptyset) = \{\emptyset\}$, i.e. a one-element set, whose only element is the empty set.
2. $\mathcal{P}(\{x\}) = \{\emptyset, \{x\}\}$, i.e. a two-element set containing the empty set and the singleton set containing x.
3. $\mathcal{P}(\{x, y\}) = \{\emptyset, \{x\}, \{y\}, \{x, y\}\}$.

4. $\mathcal{P}(\{x, y, z\}) = \{\emptyset, \{x\}, \{y\}, \{z\}, \{x, y\}, \{x, z\}, \{y, z\}, \{x, y, z\}\}$.

Remark 3. The power set of every set X contains the empty set and X.

Definition 4 (Cartesian product)**.** The *Cartesian product* of sets A and B is the set of pairs of elements $A \times B = \{(a, b) | a \in A, b \in B\}$, together with two maps, called *projections*, returning the first and second components of each pair, $\pi_A : (a, b) \mapsto a$ and $\pi_B : (a, b) \mapsto b$.

Remark 5. The Cartesian product of a set X with the empty set is the empty set, i.e. $X \times \emptyset = \emptyset = \emptyset \times X$.

Example 6 (deck of cards)**.** A deck of (52) cards corresponds to the Cartesian product of the set of ranks, $R = \{2, 3, \ldots, 10, J, Q, K, A\}$ and the set of suits, $S = \{\spadesuit, \clubsuit, \diamondsuit, \heartsuit\}$, i.e. the set $D = R \times S = \{(2, \clubsuit), (3, \clubsuit), \ldots, (A, \heartsuit)\}$.

Definition 7 (disjoint union)**.** The *disjoint union* of sets A and B is the set of pairs such that each pair consists of an element from A or an element from B paired with a label to distinguish their set of origin, $A + B = \{(1, a) | a \in A\} \cup \{(2, b) | b \in B\}$, together with two maps, called *injections*, $\iota_A : a \mapsto (1, a)$ and $\iota_B : b \mapsto (1, b)$.

Example 8 (deck of cards with joker)**.** The joker does not have a suit, but can be added to the deck of cards by disjoint union, i.e. $D + \{\mathfrak{J}\} = \{(1, (2, \clubsuit)), \ldots, (1, (A, \heartsuit)), (2, \mathfrak{J})\}$.

Remark 9. In general, set union and disjoint union are not equivalent—have the same number of elements: e.g., $\{a, b\} \cup \{b, c\} = \{a, b, c\}$, whereas $\{a, b\} + \{b, c\} = \{(1, a), (1, b), (2, b), (2, c)\}$.

Definition 10 (basic functions)**.** The following basic functions are defined.

1. An *injection* is a function $f : X \to Y$ such that $f(x) = f(x') \Rightarrow x = x'$.
2. A *surjection* is a function $f : X \to Y$ such that every element $y \in Y$ is the image of an element $x \in X$.
3. A *bijection* is a function that is an injection and a surjection.
4. The *identity function* on a set X is the map $1_X : X \to X; x \to x$.
5. A *left-inverse* to a function $f : X \to Y$ is a function $r : Y \to X$ such that $r \circ f = 1_X$.
6. A *right-inverse* to a function $f : X \to Y$ is a function $s : Y \to X$ such that $f \circ s = 1_Y$.
7. An *inclusion* of sets $X \subseteq Y$ is the function $\iota : X \to Y; x \mapsto x$.
8. A *point* of a set X is a function from a one-element set picking out an element $x \in X$, written in full as $\overline{x} : 1 \to X; * \mapsto x$, also simply denoted x and called an *element* of X.

Remark 11. There is a one-to-one correspondence between the elements x and the points $\overline{x}$ of a set X.

Definition 12 (image, preimage)**.** Suppose a function $f : X \to Y$. The *image* of a set $U \subseteq X$ under f, written $f[U]$, is the set of elements obtained by f of an element in U, i.e. $f[U] = \{y | y = f(x), x \in U\}$. The *preimage* of a set $U \subseteq Y$, written $f^{-1}[U]$, is the set of elements in X that map to an element in U, i.e. $f^{-1}[U] = \{x | y = f(x), y \in U\}$. If U is a one-element set, $\{x\} \subseteq X$, respectively, $\{y\} \subseteq Y$, then the image is written $f[x]$, respectively, the preimage is written $f^{-1}[y]$.

Definition 13 (graph of a function)**.** The *graph* of a function $f : X \to Y$ is the set of ordered pairs $\Gamma(f) = \{(x, f(y) | x \in X\} \subseteq X \times Y$.

Definition 14 (restriction)**.** Suppose a function $f : X \to Y$ and a subset U of X. The *restriction* of f to U is the function $f|_U : U \to Y$ with graph $\Gamma(f|_U) = \{(x, f(x)) | x \in U\}$.

Definition 15 (index)**.** Suppose I and S are sets. An *indexing function* is a function $f : I \to S$ that provides a reference for each element $s \in S$ by an element $i \in I$, denoted s_i. Set I is called the *index set* and S is called the *indexed set*. An *I*-indexed set S is called a *family*, denoted as the set $\{s_i\}_{i \in I}$, or the ordered tuple $(s_i)_{i \in I}$ if I has an order.

Definition 16 (function space)**.** The set of functions from X to Y is called the *function space*, denoted Y^X.

Remarks 17. The number of functions in Y^X is $|Y|^{|X|}$ where $|X|$ and $|Y|$ are the number of elements in each set. Some special cases are noted.

- The number of functions from the empty set to a set Y, i.e. in the function space $Y^{\emptyset}$, is $|Y|^0 = 1$. That is the function $f : \emptyset \to Y$ whose graph is the empty set, $\Gamma(f) = \emptyset$.
- The number of functions from any set X to a one-element set, denoted $1 = \{*\}$, is $1^{|X|} = 1$. There is just one such function, which sends every element $x \in X$ to the only element in $\{*\}$.
- There are no functions to the empty set, i.e. $0^{|X|} = 0$, except when X is also the empty set, $0^0 = 1$, which is the empty function.

Definition 18 (topological space)**.** A *topological space* is a pair (X, T) consisting of a set X and a set T of subsets of X, called the *open sets* of T, such that:

- the empty set and X are open sets, i.e. $\emptyset, X \in T$,
- arbitrary unions of open sets of T are open sets and
- finite intersections of open sets of T are open sets.

T is called the *topology* of X, also denoted $\mathcal{O}_X$. A topological space is also simply denoted X or T.

Example 19 (discrete topological space)**.** A *discrete topological space* X has all subsets of X as open sets, i.e. $(X, \mathcal{P}(X))$.

Example 20 (indiscrete topological space)**.** An *indiscrete topological space* X has the empty set and X as the only open sets. An indiscrete topology is also called a *trivial topology*.

Remarks 21. There are two topological spaces that are both discrete and indiscrete:

- the empty space, $(\emptyset, \{\emptyset\})$ and
- the one-point space, $(\{*\}, \{\emptyset, \{*\}\})$.

A space can be neither discrete, nor indiscrete, e.g., $(\{x, y\}, \{\emptyset, \{x\}, \{x, y\}\})$. A *non-discrete space* is an indiscrete space or a space that is not a discrete space.

Definition 22 (courser/finer topology)**.** Suppose a set X equipped with two topologies $T_1 \subseteq T_2$. The topology T_1 is called a *courser topology* than T_2; conversely, T_2 is called a *finer topology* than T_1.

Remark 23. The discrete space on X is always finer than the indiscrete space on X, since every space must include the empty set and X as open sets (by definition 18).

Definition 24 (continuous function)**.** Suppose topological spaces X and Y. A *continuous function* is a function $f : X \to Y$ such that for every open set U of Y the preimage $f^{-1}[U]$ is an open set of X.

Definition 25 (relation)**.** A *relation* on a set A is a subset R of the Cartesian product of A with itself, i.e. a set $R \subseteq A \times A$, which comes with two projections retrieving the first and second element of each pair, i.e. $\pi_1 : (a_1, a_2) \mapsto a_1$ and $\pi_2 : (a_1, a_2) \mapsto a_2$.

Remark 26. More generally, a relation R between sets A and B is a set $R \subseteq A \times B$ together with the projections $\pi_A : R \to A$ and $\pi_B : R \to B$.

Examples 27 (preorder, partial order)**.** Two important order relations are defined.

- A *preorder* is a relation $\leq$ on a set P that satisfies:
  - reflexivity: $p \leq p$, and
  - transitivity: $p \leq q \wedge q \leq r \Rightarrow p \leq r$

  for all triples of elements $p, q, r \in P$.
- A *partial order* is a preorder that satisfies:
  - asymmetry: $p \leq q \wedge q \leq p \Rightarrow p = q$.

  for all pairs of elements $p, q \in P$.

Every partial order is a preorder.

Definition 28 (ordered set)**.** A *preordered set (proset)*, respectively, a *partially ordered set (poset)* is a pair $(P, \leq)$ consisting of a set P and a preorder, respectively, partial order, $\leq$, on P.

Example 29 (proset)**.** Suppose the group (set) of people $\{\mathrm{John}, \mathrm{Mary}, \mathrm{Sue}\}$ are ordered by age: 35, 35, 41, respectively. We have a proset, but not a poset, since John is not older than Mary and Mary is not older than John, but John and Mary are not the same person, i.e. $\mathrm{John} \leq \mathrm{Mary} \wedge \mathrm{Mary} \leq \mathrm{John} \not\Rightarrow \mathrm{John} = \mathrm{Mary}$. However, if ordered by name, then we have a poset; in fact, a *totally ordered set*—every pair of elements $p, q \in P$ is comparable: either $p \leq q$ or $q \leq p$.

Example 30 (topology—poset)**.** A topology T is a poset of open sets, $(T, \subseteq)$, i.e. $V \leq U$ when $V \subseteq U$.

Definition 31 (monotonic function)**.** Suppose prosets $(P, \leq)$ and $(Q, \sqsubseteq)$. A *monotonic function* is a (order-preserving) function $f : P \to Q$ such that $p \leq p' \Rightarrow f(p) \sqsubseteq f(p')$ for all $p, p' \in P$.

2.2 Categories

Definition 32 (category)**.** A *category* $\mathbf{C} = (\mathbf{C}_0, \mathbf{C}_1, \circ, \mathit{dom}, \mathit{cod}, \mathit{id})$ consists of:

- a collection $\mathbf{C}_0$ of entities, called *objects*, $A, B, \ldots, X, Y, \ldots,$
- a collection $\mathbf{C}_1$ of "directed relations" between objects, called *arrows* (*maps* or *morphisms*), written $f : X \to Y$ to indicate that arrow f goes from object X to object Y, called the *domain* and *codomain* of f, respectively,
- a map $\mathit{dom} : \mathbf{C}_1 \to \mathbf{C}_0$ sending each arrow $f : X \to Y$ to its domain object, i.e. $\mathit{dom}(f) = X$,
- a map $\mathit{cod} : \mathbf{C}_1 \to \mathbf{C}_0$ sending each arrow $f : X \to Y$ to its codomain object, i.e. $\mathit{dom}(f) = Y$, and
- a map $\mathit{id} : \mathbf{C}_0 \to \mathbf{C}_1$ assigning the arrow $1_X : X \to X$, called the *identity arrow* at object X

that together satisfy:

- unity: $f \circ 1_X = f = 1_X \circ f$ for each arrow $f : X \to Y$ in $\mathbf{C}$ and
- associativity: $h \circ (g \circ f) = (h \circ g) \circ f$ for each triple of arrows f, g and h in $\mathbf{C}$.

The collection of arrows with domain A and codomain B is called a *hom-set*, written $\mathrm{Hom}(X, Y)$.

Remarks 33. Category theory concepts are typically abstractions of more familiar concepts in other branches of mathematics, such as set theory and graph theory, hence the notation is often taken from there. Some comparisons with more familiar concepts follow.

- In the context of sets and functions between sets, an arrow $f : X \to Y$ is a function from set X to set Y and a hom-set $\mathrm{Hom}(X, Y)$ is the function space Y^X. An identity arrow 1_X is the identity function $1_X : x \mapsto x$ and composition is composition of functions, i.e. $g \circ f(x) = g(f(x))$.
- A (directed) graph $G = (G_0, G_1, \mathit{src}, \mathit{tgt})$ is a set of vertices, G_0, a set of edges, G_1, and two maps $\mathit{src}, \mathit{tgt} : G_1 \to G_0$ specifying the source and target vertex of each edge, which almost constitutes a category, i.e. without necessarily having all loops (identities) and paths as edges (composition).
- In the context of order relationships, the domain may be regarded as coming "before" the codomain, i.e. an arrow $f : X \to Y$ as the order relationship $X \leq_f Y$.
- Nothing is said about the nature of objects beyond their relationships to morphisms. Hence, an object may be a set, an element of a set, or some more complex entity such as a graph or even another category. In such cases, the notation for object is often taken from that context, e.g., lower case symbols to highlight objects as elements of some set, such as an arrow $x \to y$ for $x \leq y$.
- Nothing is said about the nature of arrows beyond their relationships to objects, other arrows and the composition operation. Hence, an arrow may be a function, an order relationship or some other more complex relationship that "preserves" some of the "internal structure" of an object (i.e. relations between the parts of an object), such as a graph homomorphism. In such cases, the notation for arrows may also reflect that context, e.g., the order symbol $\leq$ in place of the usual symbol $\to$ for arrow.
- A similar remark applies to composition: e.g., in the context of order, the operation is conjunction and order composition is transitivity, cf. $(y \leq z) \wedge (x \leq y) \Rightarrow (x \leq z)$ with $(y \to z) \circ (x \to y) \mapsto (x \to z)$.

Any collections of entities that satisfy the above requirements (definition 32) is a category, hence the wide applicability of category theory.

Examples 34 (category)**.** The following are categories.

1. The *empty category*, 0, has no objects and no arrows.
2. The *singleton category*, 1, has one object and one (identity) arrow.
3. The *pair category*, 2, has two objects and no non-identity arrows.
4. The arrow category, $\vec{2}$, has two objects and one (non-identity arrow), $0 \to 1$.
5. A preordered set $(P, \leq)$ is a category whose objects are the elements $p \in P$ and arrows are the order relations, i.e. there is an arrow $p \to q$ whenever $p \leq q$.
6. A topological space (X, T) is a category whose objects are the open sets $U \in T$ and arrows are the inclusions $U \subseteq V$.
7. $\mathbf{Set}$ is the category of sets and functions.
8. $\mathbf{Set}^{\subseteq}$ is the category of sets and inclusions.

Remark 35. $\mathbf{Set}^{\subseteq}$ is a *subcategory* of $\mathbf{Set}$—a subcategory of a category $\mathbf{C}$ is a category $\mathbf{B}$ with objects and arrows from $\mathbf{C}$ (cf. subset), also written $\mathbf{B} \subseteq \mathbf{C}$.

Definition 36 (basic arrows)**.** The following basic arrows are defined in a category $\mathbf{C}$.

1. A *monomorphism* is an arrow $f : X \to Y$ such that $f \circ g = f \circ h$ implies $g = h$ for every pair of arrows $g, h : Z \to X$ in $\mathbf{C}$.

2. An *epimorphism* is an arrow $f : X \to Y$ such that $g \circ f = h \circ f$ implies $g = h$ for every pair of arrows $g, h : Y \to Z$ in $\mathbf{C}$.
3. A *retraction* of an arrow $f : X \to Y$ is an arrow $r : Y \to X$ such that $r \circ f = 1_X$.
4. A *section* of an arrow $f : X \to Y$ is an arrow $s : Y \to X$ such that $f \circ s = 1_Y$.
5. A *isomorphism* is an arrow $f : X \to Y$ that has a retraction and a section.

A retraction is also called a *left-inverse* and a section is also called a *right-inverse*, hence an isomorphism is an arrow that has both a left-inverse and a right-inverse. If $f : X \to Y$ is an isomorphism, then Y is said to be *isomorphic* to X, written $X \cong Y$.

Example 37 (basic arrows)**.** In $\mathbf{Set}$, monomorphisms are injections, epimorphisms are surjections, retractions are left-inverses, sections are right-inverses, and isomorphisms are bijections.

Definition 38 (functor)**.** A *functor* $F : \mathbf{C} \to \mathbf{D}$ is a map sending each object X in $\mathbf{C}$ to the object $F(X)$ in $\mathbf{D}$ and each arrow $f : X \to Y$ in $\mathbf{C}$ to the arrow $F(f) : F(X) \to F(Y)$ such that:

- identity: $F(1_X) = 1_{FX}$ for each object X in $\mathbf{C}$ and
- compositionality: $F(g \circ_{\mathbf{C}} f) = F(g) \circ_{\mathbf{D}} F(f)$ for each pair of arrows f and g in $\mathbf{C}$.

The collection of objects/arrows obtained from F is called the *image* of F (cf. definition 12).

Example 39 (monotone)**.** A monotonic function is a functor between prosets as categories (cf. definition 31): the identity condition is given by $p \leq p \Rightarrow f(p) \sqsubseteq f(p)$ and compositionality by $p \leq q \Rightarrow f(p) \sqsubseteq f(q)$.

Example 40 (diagram)**.** A *diagram* of shape J in a category $\mathbf{C}$ is a functor $D : J \to \mathbf{C}$ that acts as the categorical analog of an indexed set (cf. definition 15) by picking out a (J-)collection of objects and arrows in $\mathbf{C}$. For instance, a pair of objects A and B in $\mathbf{C}$ corresponds to the diagram $(A, B) : 2 \to \mathbf{C}$.

Remarks 41. The concept "shape" is used in several closely related ways: as the shape of

1. a diagram $D : J \to \mathbf{C}$, i.e. the domain category J,
2. an arrow $f : X \to Y$, i.e. the domain object X, and
3. a topological space X for a functor $\mathcal{F} : X^{\mathrm{op}} \to \mathbf{Set}$, called a *presheaf* (next section), i.e. the topology of the domain (space) X.

Definition 42 (natural transformation, equivalence)**.** Suppose functors $F, G : \mathbf{C} \to \mathbf{D}$. A *natural transformation* written $\eta : F \dot{\to} G$ is a family of $\mathbf{D}$-arrows $\eta = \{\eta_X : F(X) \to G(X)\}_{X \in \mathbf{C}_0}$ such that $\eta_Y \circ F(f) = G(f) \circ \eta_X$ for each $\mathbf{C}$-arrow $f : X \to Y$, as indicated by the following diagram:

$$\begin{array}{ccccc} X & & F(X) & \xrightarrow{\eta_X} & G(X) \\ {\scriptstyle f}\downarrow & & {\scriptstyle F(f)}\downarrow & & \downarrow{\scriptstyle G(f)} \\ Y & & F(Y) & \xrightarrow[\eta_Y]{} & G(Y) \end{array} \tag{S1}$$

A map η_X is called the *component* of η at X. If every component is an isomorphism, then the natural transformation is called a *natural equivalence*. The "square" of arrows is called a *commutative square*.

Example 43 (diagram homomorphism)**.** Diagrams are functors; maps between diagrams (i.e. diagram homomorphisms) are natural transformations. For example, an arrow-shaped diagram in a category $\mathbf{C}$ is a functor $\alpha : \vec{2} \to \mathbf{C}$ that picks out an arrow $\alpha : A_0 \to A_1$ in $\mathbf{C}$. Accordingly, an arrow homomorphism is a

natural transformation $\phi : \alpha \dot{\rightarrow} \beta$, i.e. a pair of arrows $(\phi_0 : A_0 \rightarrow B_0, \phi_1 : A_1 \rightarrow B_1)$, both in $\mathbf{C}$, such that the following diagram commutes:

$$\begin{array}{ccc} A_0 & \xrightarrow{\phi_0} & B_0 \\ {\scriptstyle\alpha}\big\downarrow & & \big\downarrow{\scriptstyle\beta} \\ A_1 & \xrightarrow[\phi_1]{} & B_1 \end{array} \tag{S2}$$

Remark 44. The collection of functors from a category $\mathbf{C}$ to a category $\mathbf{D}$ and their natural transformations constitute a *functor category*, denoted $\mathbf{D}^{\mathbf{C}}$ (cf. function space, definition 16). The objects of $\mathbf{D}^{\mathbf{C}}$ are the functors $F : \mathbf{C} \rightarrow \mathbf{D}$ and the arrows are the natural transformations $\eta : F \dot{\rightarrow} G$.

Example 45 (diagrams)**.** A collection of *J*-shaped diagrams in a category $\mathbf{C}$ is the functor category $\mathbf{C}^J$. For instance, the category of arrows in $\mathbf{C}$ is $\mathbf{C}^{\vec{2}}$, also denoted $\mathbf{Arr}(\mathbf{C})$.

Remarks 46. A diagram acts like directed attention to some region within a field of view, hence a diagram homomorphism acts like a shift of attention:

$$\begin{array}{ccccc} & & J & & \\ & \swarrow & & \searrow & \\ D(J) & & \xrightarrow[\sigma]{} & & D'(J) \end{array} \tag{S3}$$

where J is a collection of pointers and $D(J)$ is the collection of items attended to (and their relations).

Example 47 (diagonal)**.** The diagonal functor $\Delta : \mathbf{C} \rightarrow \mathbf{C}^J$ sends each object X in $\mathbf{C}$ to the (*J*-shaped) diagram $X : J \rightarrow \mathbf{C}$, i.e. the (constant) functor $\Delta(X)$ sending every object and arrow in J to the same object X and identity arrow 1_X in $\mathbf{C}$, and each arrow $f : X \rightarrow Y$ in $\mathbf{C}$ to the natural transformation between diagrams $\phi_f : X \dot{\rightarrow} Y$, i.e. the (constant) family of $\mathbf{C}$-arrows $\phi_f = \{f : X \rightarrow Y\}_{i \in J_0}$, which commute trivially since the only arrows in the images of $\Delta(X)$ and $\Delta(Y)$ are the identities, 1_X and 1_Y. Some cases follow.

1. When $J = 2$, the diagonal functor acts like a copy process, i.e. $\Delta(X) = (X, X)$.
2. When $J = 0$, the diagonal functor is $\Delta : \mathbf{C} \rightarrow \mathbf{C}^0 \cong 1$. So, every object X and arrow f is sent to the only (unnamed) object in the singleton category, i.e., $\Delta(X) = *$, and its identity arrow.

Remarks 48. The construction of objects and arrows from other categories leads to a definition for a general class of universal construction, called *limit*.

1. In a category of *J*-shaped diagrams in a category $\mathbf{C}$, i.e. a functor category $\mathbf{C}^J$, the diagonal functor applied to an object V (called a vertex), i.e. diagram $\Delta(V)$, and an arrow from $\Delta(V)$ to a diagram D, i.e. a natural transformation $\phi : \Delta(V) \dot{\rightarrow} D$, constitutes a construction called a *cone* to D, indicated by the following commutative diagram of objects and arrows in $\mathbf{C}$:

$$\begin{array}{ccc} V & \xrightarrow{\phi_i} & D(i) \\ & {\scriptstyle\phi_j}\searrow & \big\downarrow{\scriptstyle D(ij)} \\ & & D(j) \end{array} \tag{S4}$$

(Objects i and j and arrow ij are in J.) D is called the *base* and ϕ is called the legs of the cone.

2. The cones to a fixed base D are given by the vertex and legs, i.e. the pairs (V, ϕ). A map between cones, $h : (V, \phi) \to (W, \psi)$, is called a *cone homomorphism*, i.e. an arrow h in $\mathbf{C}$ such that the following diagram commutes:

$$\begin{array}{ccc} \Delta(V) & & \\ {\scriptstyle \Delta(h)}\downarrow & \searrow^{\phi} & \\ \Delta(W) & \xrightarrow[\psi]{} & D \end{array} \tag{S5}$$

where the objects and arrows in this diagram are now in the functor category $\mathbf{C}^J$.

3. The collection of cones to a (J-shaped) diagram D and their homomorphisms constitute a category, denoted $\mathbf{Cone}(\mathbf{C}^J, D)$—the objects are the pairs (V, ϕ) and arrows are the maps $h : (V, \phi) \to (W, \psi)$. In this context (category), a special cone is defined with the following universal mapping property: a *limit* to a diagram D is a cone, denoted $(L, \lim)$, such that for every cone (V, ϕ) in $\mathbf{Cone}(\mathbf{C}^J, D)$ there exists a unique cone homomorphism $u : (V, \phi) \to (L, \lim)$, as indicated by the following diagram:

$$\begin{array}{c} (V, \phi) \\ {\scriptstyle u}\, \vdots\!\downarrow \\ (L, \lim) \end{array} \tag{S6}$$

where the objects and arrow in this diagram are now in the category $\mathbf{Cone}(\mathbf{C}^J, D)$. A dashed arrow indicates uniqueness.

4. A limit is an instance of a universal construction called a *terminal object*: an object, denoted 1, in a category $\mathbf{C}$ such that for every object Z in $\mathbf{C}$ there exists a unique arrow $u : Z \to 1$. In other words, a limit to a diagram D is a terminal object (or, *universal cone*) in the category of cones $\mathbf{Cone}(\mathbf{C}^J, D)$.

5. These constructions dualize as *cocones*, *cocone homomorphisms* and *colimits*. A colimit is an *initial object* in the category of cocones, denoted $\mathbf{Cocone}(\mathbf{C}^J, D)$—an initial object in a category $\mathbf{C}$ is an object, denoted 0, such that for every object Z in $\mathbf{C}$ there exists a unique arrow $u : 0 \to Z$. The initial object in $\mathbf{C}$ is the terminal object in $\mathbf{C}^{\mathrm{op}}$.

Definition 49 (limit, colimit)**.** A *limit* to a diagram D is a universal cone to D. Dually, a *colimit* to D is a universal cocone to D.

Example 50 (product, coproduct)**.** The product of objects A and B is the limit to the diagram (A, B) in the category of cones $\mathbf{Cone}(\mathbf{C}^2, (A, B))$; dually, the coproduct (cf. remarks 48):

1. The pair (A, B) is the base diagram in $\mathbf{C}^2$ and a cone to (A, B) is given by the following diagram:

$$\begin{array}{ccc} Z & \xrightarrow{f} & A \\ & {}_{g}\searrow & \\ & & B \end{array} \tag{S7}$$

2. the cone homomorphisms by diagram

$$\begin{array}{ccc} Z & & \\ {\scriptstyle z}\downarrow & \searrow^{(f,g)} & \\ Z' & \xrightarrow[(h,k)]{} & (A, B) \end{array} \tag{S8}$$

3. the limit by diagram

$$\begin{array}{c} (Z, (f, g)) \\ {\scriptstyle \langle f,g \rangle} \downarrow \\ (A \times B, (\pi_A, \pi_B)) \end{array} \tag{S9}$$

For the category of sets and functions, $\mathbf{Set}$, the limit is the Cartesian product and the two projections, and the colimit is the disjoint union and the two injections.

Remark 51. Limits and colimits are determined by shape, J.

1. There are four basic kinds of limits:
   a. terminal: $J = 0$, i.e. the empty category,
   b. product: $J = 2$, i.e. the two-object category with no non-identity arrows,
   c. equalizer: $J = (\cdot \rightrightarrows \cdot)$, i.e. the two-object category with two parallel arrows and
   d. pullback: $J = (\cdot \to \cdot \leftarrow \cdot)$, i.e. the three-object category with two converging arrows.
2. There are four basic kinds of colimits:
   a. initial: $J = 0$,
   b. coproduct: $J = 2$,
   c. coequalizer: $J = (\cdot \rightrightarrows \cdot)$ and
   d. pushout: $J = (\cdot \leftarrow \cdot \to \cdot)$, i.e. the three-object category with two diverging arrows.

Finite limits are constructed from terminals and pullbacks (equivalently, products and equalizers). Dually, finite colimits are constructed from initials and pushouts (equivalently, coproducts and coequalizers).

Remark 52. Finding a limit can be interpreted as an optimization process as every object in the category eventually transforms to the limit (terminal) object by following arrows in the category of cones; dually, colimits. Not all arrows need point to the limit directly, so finding the limit need not be a one-step process.

Example 53 (discrete, indiscrete topologies)**.** The discrete and indiscrete topologies are extremal in the (co)limit sense: in the category of topologies for a set X, denoted $\mathbf{Top}(X)$, whose objects are the topologies $\mathcal{O}_X$ and arrows are inclusions, the indiscrete and discrete topologies are the initial and terminal objects, respectively, i.e. $\{\emptyset, X\} \subseteq \mathcal{O}_X \subseteq \mathcal{P}(X)$ for every topology $\mathcal{O}_X$ in $\mathbf{Top}(X)$. In this situation, the shape of the diagrams is the empty category, i.e. $J = 0$, so the cones have no legs, i.e. the empty natural transformation, $\emptyset$. Since every cone in this category is a pair $(V, \emptyset)$, the limit (discrete topology) is given by the following diagram of cones as vertexes:

$$\begin{array}{c} \mathcal{O}_X \\ {\scriptstyle \subseteq} \downarrow \\ \mathcal{P}(X) \end{array} \tag{S10}$$

and dually for the colimit (indiscrete topology). We can interpret this limit process as discretization of a space, hence a process underlying a transition from non-symbolic to symbolic representations.

Remarks 54. The category (context) in which the product (limit) is a universal construction is fixed by the diagram (A, B). This view of universal construction is *internal* to the given category. Expanding context by varying the base diagram yields a functorial relationship. Two such situations follow.

1. The product (limit) functor $\Pi : \mathbf{C}^2 \to \mathbf{C}$ sends each pair of objects (A, B) to their product $A \times B$ and each pair of arrows (f, g) to their product $f \times g$. In $\mathbf{Set}$, the product function is $f \times g : (x, y) \mapsto (f(x), g(y))$.
2. The coproduct (colimit) functor $\amalg : \mathbf{C}^2 \to \mathbf{C}$ sends each pair of objects (A, B) to their coproduct $A + B$ and each pair of arrows (f, g) to their coproduct $f + g$. In $\mathbf{Set}$, the coproduct function is $f + g : (1, x) \mapsto f(x), (2, y) \mapsto g(y)$.

These functors pertain to part-whole relationships, hence logical inferences.

Example 55 (implication)**.** Logical implications $A \wedge B \Rightarrow A$ (elimination) and $A \Rightarrow A \vee B$ (expansion) have an interpretation in terms of natural transformations between (co)product and projection functors, $\acute{\Pi} : (A, B) \to A$:

1. elimination: $\acute{\pi} : \Pi \dot{\to} \acute{\Pi}$, as given by commutative diagram

$$\begin{array}{ccc} A \times B & \xrightarrow{\acute{\pi}_A} & A \\ {\scriptstyle f \times g}\downarrow & & \downarrow{\scriptstyle f} \\ A' \times B' & \xrightarrow[\acute{\pi}_{A'}]{} & A' \end{array} \tag{S11}$$

   and
2. expansion: $\acute{\iota} : \acute{\Pi} \dot{\to} \amalg$, as given by commutative diagram

$$\begin{array}{ccc} A & \xrightarrow{\acute{\iota}_A} & A + B \\ {\scriptstyle f}\downarrow & & \downarrow{\scriptstyle f+g} \\ A' & \xrightarrow[\acute{\iota}_{A'}]{} & A' + B' \end{array} \tag{S12}$$

In $\mathbf{Set}$, elimination and expansion correspond to natural projections and natural injections, respectively. In $\mathbf{Set}^{\subseteq}$, elimination corresponds to $A \cap B \subseteq A$ and expansion to $A \subseteq A \cup B$. In a poset, $(P, \leq)$, elimination corresponds to $p \wedge q \leq p$ and expansion to $p \leq p \vee q$, which pertain to the *supremum* and *infimum*, respectively, i.e. the smallest value not less than p or q is not greater than p; dually, the largest value not greater than p or q is not smaller than p. When P is the set of Boolean values, $\mathbb{B} = \{F, T\}$, ordered by implication, $F \Rightarrow F$, $F \Rightarrow T$ and $T \Rightarrow T$, elimination and expansion are just the logical forms as natural transformations. A logical equivalence, $\Leftrightarrow$, corresponds to a natural equivalence.

Example 56 (monotonicity)**.** The condition for a monotone function (example 39) is just the condition for a functor construed as a natural transformation:

$$\begin{array}{ccc}(0,p) & \xrightarrow{\phi_p} & (1,f(p)) \\ {\scriptstyle (0,\leq)}\big\downarrow & & \big\downarrow{\scriptstyle (1,\sqsubseteq)} \\ (0,q) & \xrightarrow[\phi_q]{} & (1,f(q))\end{array} \tag{S13}$$

where ϕ takes on the role of the implication.

Remark 57. Limits were presented as special kinds of objects internal to a category. An alternative view presents limits as pertaining to special kinds of arrows to/from a functor, i.e. *external* to a category. This external view affords a more general notion of universal construction, which subsumes limits.

Definition 58 (universal morphism)**.** The definition of *universal morphism* has two forms (by duality).

- Primal. A universal morphism from an object X in a category $\mathbf{C}$ to a functor $F : \mathbf{D} \to \mathbf{C}$ is a pair (A, η) consisting of an object A in $\mathbf{D}$ and an arrow $\eta : X \to F(A)$ in $\mathbf{C}$ such that for every object Y in $\mathbf{D}$ and arrow $f : X \to F(Y)$ in $\mathbf{C}$ there exists a unique arrow $u : A \to Y$ in $\mathbf{D}$ such that $f = F(u) \circ \eta$, as indicated by the following commutative diagram:

$$\begin{array}{ccccc}X & \xrightarrow{\eta} & F(A) & \qquad & A \\ & {\scriptstyle f}\searrow & \big\downarrow{\scriptstyle F(u)} & & \big\downarrow{\scriptstyle u} \\ & & F(Y) & & Y\end{array} \tag{S14}$$

- Dual. A universal morphism from a functor $F : \mathbf{C} \to \mathbf{D}$ to an object X in a category $\mathbf{D}$ is a pair (A, ϵ) consisting on an object A in $\mathbf{C}$ and an arrow $\epsilon : F(A) \to X$ in $\mathbf{D}$ such that for every object Y in $\mathbf{C}$ and arrow $f : F(Y) \to X$ in $\mathbf{D}$ there exists a unique arrow $u : Y \to A$ in $\mathbf{C}$ such that $f = \epsilon \circ F(u)$, as indicated by the following commutative diagram:

$$\begin{array}{ccccc}Y & \qquad & F(Y) & & \\ {\scriptstyle u}\big\downarrow & & {\scriptstyle F(u)}\big\downarrow & \searrow{\scriptstyle f} & \\ A & & F(A) & \xrightarrow[\epsilon]{} & X\end{array} \tag{S15}$$

Example 59 (product)**.** The *product* of a pair of objects (A, B) is universal morphism from the diagonal functor $\Delta : \mathbf{C} \to \mathbf{C}^2$ to (A, B), i.e. the pair $(A \times B, \pi)$, where $\pi = (\pi_A, \pi_B)$, as indicated by the following commutative diagram:

$$\begin{array}{ccccc}Z & \qquad & (Z,Z) & & \\ {\scriptstyle \langle f,g\rangle}\big\downarrow & & {\scriptstyle \Delta\langle f,g\rangle}\big\downarrow & \searrow{\scriptstyle (f,g)} & \\ A \times B & & \Delta(A \times B) & \xrightarrow[(\pi_A,\pi_B)]{} & (A,B)\end{array} \tag{S16}$$

where the objects/arrows forming the triangle are in $\mathbf{C}^2$.

Example 60 (coproduct)**.** The *coproduct* of a pair of objects (A, B) is universal morphism from (A, B) to the diagonal functor $\Delta : \mathbf{C} \to \mathbf{C}^2$, i.e. the pair $(A + B, \pi)$, where $\iota = (\iota_A, \iota_B)$, as indicated by the

following commutative diagram:

$$\begin{array}{ccccc} (A,B) & \xrightarrow{(\iota_A,\iota_B)} & \Delta(A+B) & & A+B \\ & \searrow^{(f,g)} & \downarrow{\scriptstyle \Delta[f,g]} & & \downarrow{\scriptstyle [f,g]} \\ & & (Z,Z) & & Z \end{array} \tag{S17}$$

Remarks 61. This view of (co)products generalizes to limits and colimits as universal morphisms.

1. A limit to a J-shaped diagram to a category $\mathbf{C}$, i.e. a functor $D : J \to \mathbf{C}$, is a universal morphism from the diagonal functor $\Delta : \mathbf{C} \to \mathbf{C}^J$ to D, is the pair (L, ϵ), as indicated by the following commutative diagram:

$$\begin{array}{ccccc} Z & & \Delta(Z) & & \\ \downarrow{\scriptstyle u} & & \downarrow{\scriptstyle \Delta(u)} & \searrow^{h} & \\ L & & \Delta(L) & \xrightarrow[\epsilon]{} & D \end{array} \tag{S18}$$

2. Dually, a colimit is a universal morphism from D to Δ, i.e. the (L, η) indicated in commutative diagram

$$\begin{array}{ccccc} D & \xrightarrow{\eta} & \Delta(L) & & L \\ & \searrow_{h} & \downarrow{\scriptstyle \Delta(u)} & & \downarrow{\scriptstyle u} \\ & & \Delta(Z) & & Z \end{array} \tag{S19}$$

Example 62 (pullback)**.** The limit to a pair of converging arrows $f : A \to C \leftarrow B : g$ is the pullback of g along f (equivalently, f along g), i.e. an object P, also denoted $A \times_C B$ and a pair of arrows (π_A, π_B) such that the following diagram commutes:

$$\begin{array}{ccc} A \times_C B & \xrightarrow{\pi_B} & B \\ {\scriptstyle \pi_A}\downarrow & & \downarrow{\scriptstyle g} \\ A & \xrightarrow[f]{} & C \end{array} \tag{S20}$$

In $\mathbf{Set}$, the pullback object $A \times_C B$ consists of the set of pairs of elements from A and B constrained to have the same image under f and g, i.e. $\{(a, b) | f(a) = f(b)\}$. If C is the terminal object, then the pullback is just the product: e.g., $f(a) = * = f(b)$ for all $(a, b) \in A \times B$.

Remark 63. For $J = 1$, i.e. the one-object category whose only arrow is the identity on that object, the limit to A is any object B isomorphic to A and the isomorphism, i.e. $B \cong A$. In $\mathbf{Set}$, the limit to set A is any set B in bijective correspondence with A and the bijection.

Definition 64 (adjunction)**.** Suppose $\mathbf{C}$ and $\mathbf{D}$ are categories. An *adjunction* from $\mathbf{C}$ to $\mathbf{D}$, written $(F, G, \eta, \epsilon, \phi, \psi) : \mathbf{C} \rightharpoonup \mathbf{D}$, consists of the following data:

- a pair of (opposing) functors $F : \mathbf{C} \to \mathbf{D}$ and $G : \mathbf{D} \to \mathbf{C}$, called the *left adjoint* and the *right adjoint*, respectively,
- a pair of natural transformations $\eta : 1_{\mathbf{C}} \dot{\to} G \circ F$ and $\epsilon : G \circ F \dot{\to} 1_{\mathbf{D}}$, called the *unit* and the *counit* of the adjunction, respectively, and

- a pair of bijections $\phi_{X,Y} : [FX, Y] \to [X, GY]$ and $\psi_{X,Y} : [X, GY] \to [FX, Y]$, called the *left adjunct* and the *right adjunct*, respectively, for each pair of objects (X, Y) in $\mathbf{C} \times \mathbf{D}$

such that for every object X in $\mathbf{C}$ there is a universal morphism from X to G, i.e. the pair (FX, η_X); equivalently, for every object Y in $\mathbf{D}$ there is a universal morphism F to Y, i.e. the pair (GY, ϵ_Y). The relationship between adjoints F and G is called an *adjoint situation*, written $F \dashv G$.

Remark 65. The relationships between these data are indicated by the following diagrams for

- the unit:

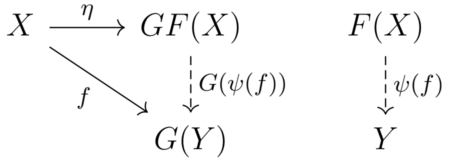

(S21)

and

- the counit

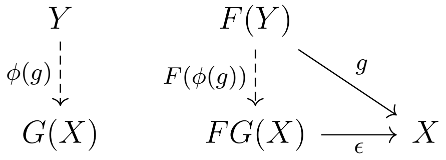

(S22)

of the adjunction. Adjoints are universals (cf. diagrams S21 and S14, and diagrams S22 and S15).

Definition 66 (equivalence of categories)**.** An *equivalence of categories* $\mathbf{C}$ and $\mathbf{D}$ is an adjoint situation $(F, G, \eta, \epsilon) : \mathbf{C} \rightharpoonup \mathbf{D}$ such that η and ϵ are natural equivalences. In this situation, $\mathbf{D}$ is said to be *equivalent* to $\mathbf{C}$, written $\mathbf{C} \simeq \mathbf{D}$.

Remark 67. Universal constructions, including (co)limits, are *unique up to a unique isomorphism*. There may be more than one universal construction for a given situation but there is only one map between two such constructions. For instance, another product of A and B is $B \times A$, which has the order of elements systematically swapped, together with the appropriate projections. Conventionally, $A \times B$ is given as *the* product and so is sometimes called the *canonical product* and likewise for other universal constructions.

Definition 68 (exponential)**.** The product functor $\Pi_B : \mathbf{C} \to \mathbf{C}$ sends each object A to the product with B, i.e. $A \times B$, and each arrow f to the product with the identity on B, i.e. $f \times 1_B$. The *exponential* functor $\Lambda_B : \mathbf{C} \to \mathbf{C}$ sends each object A to the exponential object A^B. These functors are adjoints, $\Pi_B \dashv \Lambda_B$. The counit of the adjuntion, ϵ, is given by the following commutative diagram:

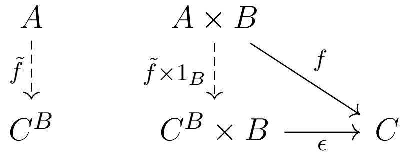

(S23)

$\tilde{f}$ is called the transpose of f and pair (C^B, ϵ) is called the *exponential object*.

Example 69 (evaluation)**.** In **Set**, the counit is called *evaluation*, written $eval$—the transpose $\tilde{f}$ takes value $a \in A$ and returns the function ϕ_a in function space C^B and $eval$ evaluates ϕ_a at value $b \in B$, i.e. $eval(f_a, b) = f(a, b)$. This situation is called the *curry-uncurry transpose* in computer science, which is used to covert between (bivariate) function and (unary) operator: e.g., $(+_a)b = +(a, b)$, i.e. $a + b$.

Remark 70. The exponential object is a universal morphism from Π_B to C, i.e. the pair (C^B, ϵ), so generalizes the concept of limit by replacing the diagonal functor with the product functor.

Definition 71 (Kan extension)**.** Suppose categories $\mathbf{A}$, $\mathbf{B}$ and $\mathbf{C}$ and functors $X : \mathbf{A} \to \mathbf{C}$ and $F : \mathbf{A} \to \mathbf{B}$, as shown in the following diagram:

$$\begin{array}{ccccc} & & \mathbf{B} & & \\ & \overset{F}{\nearrow} & & \underset{R}{\overset{L}{\searrow\!\!\searrow}} & \\ \mathbf{A} & & \xrightarrow[X]{\qquad} & & \mathbf{C} \end{array} \tag{S24}$$

There are two forms of Kan extension.

- Right. The *right Kan extension* of X along F is a pair (R, ϵ) consisting of a functor $R : \mathbf{B} \to \mathbf{C}$ (diagram S24) and a natural transformation $\epsilon : R \circ F \dot{\to} X$ such that for every functor $M : \mathbf{B} \to \mathbf{C}$ and natural transformation $\mu : M \circ F \dot{\to} X$ there exists a unique natural transformation $\delta_F : M \dot{\to} R$ such that $\mu = \epsilon \circ \delta_F$, as indicated by the following commutative diagram:

$$\begin{array}{ccc} MF & & \\ {\scriptstyle \delta_F}\big\downarrow & \overset{\mu}{\searrow} & \\ RF & \xrightarrow[\epsilon]{} & X \end{array} \tag{S25}$$

  R is also denoted $\mathrm{Ran}_F X$.
- Left. The *left Kan extension* of X along F is a pair (L, η) consisting of a functor $L : \mathbf{B} \to \mathbf{C}$ (diagram S24) and a natural transformation $\eta : L \circ F \dot{\to} X$ such that for every functor $N : \mathbf{B} \to \mathbf{C}$ and natural transformation $\nu : X \dot{\to} N \circ F$ there exists a unique natural transformation $\delta_F : L \dot{\to} N$ such that $\nu = \delta_F \circ \eta$, as indicated by the following commutative diagram:

$$\begin{array}{ccc} X & \xrightarrow{\eta} & LF \\ & \underset{\nu}{\searrow} & \big\downarrow{\scriptstyle \delta_F} \\ & & NF \end{array} \tag{S26}$$

  L is also denoted $\mathrm{Lan}_F X$.

Remarks 72. Kan extensions are universal constructions (cf. diagrams S26 and S14, and S25 and S15). The names for right and left Kan extensions are swapped for some authors, as noted here (Mac Lane, 1998).

Example 73 (limit functor)**.** The limit of a *J*-shaped diagram in $\mathbf{C}$ is the right Kan extension of the diagonal functor Δ along the functor !, as indicated by the following diagram:

$$\begin{array}{ccccc} & & 1 & & \\ & \overset{!}{\nearrow} & & \overset{\lim}{\searrow} & \\ J & & \xrightarrow[\Delta]{\qquad} & & \mathbf{C}^J \end{array} \tag{S27}$$

The colimit is the corresponding left Kan extension. For instance, the right (left) Kan extension of the 2-shaped diagram (A, B) in $\mathbf{Set}$ is the Cartesian product (disjoint union) of sets A and B.

Example 74 (adjoint functor)**.** The right (left) adjoint to a functor $F : \mathbf{C} \to \mathbf{D}$ is the right (left) Kan extension of the identity functor $1_{\mathbf{C}}$ along F, as indicated by the following diagram:

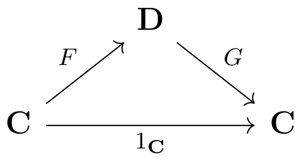

(S28)

For instance, the right (left) adjoint to the diagonal functor $\Delta : \mathbf{Set} \to \mathbf{Set}^2$ is the product (coproduct) functor sending each pair of sets to their Cartesian product (disjoint union).

2.3 Presheaves

Definition 75 (presheaf—set-theoretic version)**.** Suppose a topological space (X, T). A *presheaf* is a map $\mathcal{F} : T^{\text{op}} \to \mathbf{Set}$ sending each open set U in T to the set of elements $\mathcal{F}(U)$, called the *sections* of U and each inclusion $V \subset U$ to a function, written $res_{V,U} : \mathcal{F}(U) \to \mathcal{F}(V)$, called the *restriction morphism*, that satisfies the following conditions:

- identity: $res_{U,U} : \mathcal{F}(U) \to \mathcal{F}(U)$ is the identity function on $\mathcal{F}(U)$ for each on open set U in T and
- compositionality: if $W \subseteq V \subseteq U$, then $res_{W,V} \circ res_{V,U} = res_{W,U}$ for all open sets U, V, W in T.

An element of $\mathcal{F}(U)$ is called a *section* of U. A section of X is called a *global section*.

Remarks 76. Some remarks on the definition.

- In general, the sections of an open set need not be right-inverses (see definition 10).
- A restriction morphism $res_{V,U}$ is also written $f|_V$ (cf. function restriction, definition 14).
- The conditions for a presheaf are just the conditions for a functor (cf. definition 38).

Definition 77 (presheaf—category-theoretic version)**.** A *presheaf* is a functor $\mathcal{F} : T^{\text{op}} \to \mathbf{Set}$.

Remark 78. The move from presheaf to sheaf is about whether the sections of an open set U can be recovered from restrictions to smaller sets U_i of an *open cover* $\{U_i\}_{i \in I}$ containing U, i.e. $U \subseteq \bigcup_{i \in I} U_i$.

Example 79 (relational table)**.** The presheaf corresponding to a binary relation can be expressed in terms of relational database tables whereby column names correspond to the elements of the topological space with the discrete topology and the rows to sections of the corresponding open sets. For instance, the relationships *John loves Mary* and *Sue loves Tom* and corresponding presheaf are specified by the following table:

Sue	Tom
John	Mary
Subject	Object

The open sets $\{\text{Subject}\}$ and $\{\text{Object}\}$ and their sections are given by corresponding one-column tables. The empty set corresponds to the empty table, i.e. a table with no columns and a single (dummy) row.

Remark 80. The relational database view is a special case of presheaves in that the topological spaces are discrete spaces and the restrictions are projections affording access to all constitutents of all rows, as desired for a relational system. Presheaves have more general applicability to situations where the spaces need not be discrete, the sections need not be right-inverses and the restrictions need not be projections.

Example 81 (associational table)**.** Associations are typically in one direction: e.g., bread is associated to butter, but butter does not generally associate to bread. This “unidirectionality” can be expressed as a presheaf of a non-discrete topological space as illustrated with the following table of associations:

Cue	Associate
bread	butter
knife	fork

The roles of cue and associate are given by the set $\{C, A\}$ with the (non-discrete) topology $\{\emptyset, \{A\}, \{C, A\}\}$. In this situation, the sections on the open set $\{C, A\}$ (and the open set $\{C\}$) are just the cues, i.e. the set $\{\mathrm{bread}, \mathrm{knife}\}$. Unidirectionality is captured by the inclusion $\{A\} \subseteq \{C, A\}$ which is sent to the restriction morphism from cues to associates: e.g., $\mathrm{bread} \mapsto \mathrm{butter}$. Because $\{C\}$ is not an open set, there is no restriction morphism from associates to cues.

Definition 82 (sheaf—set-theoretic version)**.** A *sheaf* is a presheaf $\mathcal{F} : T^{\mathrm{op}} \to \mathbf{Set}$ that satisfies

- locality (uniqueness): if $\{U_i\}_{i \in I}$ is an open cover of an open set $U \in T$ and if s and t are sections of U such that $s|_{U_i} = t|_{U_i}$, then $s = t$ and
- compatibility/gluing (existence): if $\{U_i\}_{i \in I}$ is an open cover of an open set $U \in T$ and if for each pair of sections (s_i, s_j) for each pair of open sets (U_i, U_j), $i, j \in I$ (i.e. for every section s_i of U_j and for every section s_j of U_j) that agree on their overlaps, i.e. $s_i|_{U_i \cap U_j} = s_j|U_i \cap U_j$ for every $i, j \in I$, then there exists a section s on U such that $s|_{U_i} = s_i$ and $s|_{U_j} = s_j$.

Remark 83. The locality and compatibility conditions are independent.

- A presheaf that satisfies locality is called a *separated presheaf.*
- A presheaf that satisfies compatibility is called a *compatible presheaf.*

A presheaf that satisfies locality and compatibility is a sheaf and a presheaf is that does not satisfy either condition is just a presheaf.

Examples 84 (separable, compatible)**.** The following examples illustrate these conditions.

1. The loves relation (example 79) is a separable but not compatible presheaf, hence not a sheaf, because it fails to satisfy the gluing condition. For instance, Sue is a section of the open set $\{\mathrm{Subject}\}$ and Mary is a section of the open set $\{\mathrm{Object}\}$ that agree on restriction to their intersection, i.e. they both restrict to the section $*$ on the empty set, but the pair (Sue, Mary) is not a section of the open set $\{\mathrm{Subject}, \mathrm{Object}\}$. In other words, Sue is a lover and Mary is loved, but Sue is not a lover of Mary.
2. Suppose a different loves relation whereby there are two different people called John who both love the same Mary: e.g., *John (Smith) loves Mary* and *John (Doe) loves Mary*. This situation is a compatible but not separable presheaf, since the sections of the each open set are the same (by first name) but are not the same people, hence cannot be separated with this data. The locality condition is analogous to the condition for being an injective function (cf. definition 10).
3. A separable and compatible presheaf, i.e. a sheaf, occurs when all lovers and loves: e.g., the presheaf corresponding to *John loves Mary*, *Mary loves John*, J*ohn loves John* and *Mary loves Mary*.

Remark 85. The conditions for a sheaf are the unique-existence conditions for limits, which affords a more general notion of sheaf for categories other than $\mathbf{Set}$.

Definition 86 (sheaf—category-theoretic version)**.** Suppose a category $\mathbf{C}$ with finite limits. A *sheaf* is a presheaf $\mathcal{F} : T^{\mathrm{op}} \to \mathbf{C}$ such that the following diagram:

$$\mathcal{F}(U) \longrightarrow \prod_i \mathcal{F}(U_i) \rightrightarrows \prod_{i,j} \mathcal{F}(U_i \cap U_j) \tag{S29}$$

is an equalizer for every open cover $\{U_i\}_{i \in I}$ for every open set U in T.

Remark 87. A sheaf is a kind of universal construction for a presheaf. The move from separated presheaf to sheaf acts like completion of a (multiplication) table. The move from compatible presheaf to sheaf acts like removal (division) of replicates.

Example 88 (sheaf of sections)**.** Every continuous function $f : Y \to X$ induces a *sheaf of sections*, denoted $\Gamma(Y/X)$—the sections of each open set $U \subseteq X$ are the right inverses s of f restricted to U, i.e. $\Gamma(Y/X) : U \mapsto \{s | f \circ s = 1_U\}$, where $s : U \to X$.

Example 89 (playing cards)**.** To play a game of cards, one needs to recognize the suit of each card, i.e. a map from faces to suits, e.g., $suit : 2\heartsuit \mapsto \heartsuit$. As a continuous function, this map induces the sheaf of sections recovering the set of cards for each suit, e.g. $\mathcal{F}_{\mathrm{suit}} : \heartsuit \mapsto \{2\heartsuit, \ldots, A\heartsuit\}$.

Definition 90 (stalk)**.** Suppose a topological space (X, T), a presheaf $\mathcal{F} : T^{\mathrm{op}} \to \mathbf{Set}$ and an element $x \in X$. The *stalk* of $\mathcal{F}$ at x, denoted $\mathcal{F}_x$, is the direct limit $\mathcal{F}_x = \varinjlim_{U \ni x} \mathcal{F}(U)$, i.e. the limit of sections on smaller open sets containing x.

Remark 91. Information about a sheaf may be obtained from information about its stalks.

Example 92 (cards)**.** Stalks act like dimensions of a space. For instance, a deck of cards as the sheaf on $\{\mathrm{suit}, \mathrm{rank}\}$ with the discrete topology can be reconstructed from the set of suits and the set of ranks given by the stalks $\mathcal{F}_{\mathrm{suit}}$ and $\mathcal{F}_{\mathrm{rank}}$.

Definition 93 (germ)**.** Suppose a presheaf $\mathcal{F} : X^{\mathrm{op}} \to \mathbf{Set}$ and two open sets U and V that contain a point $x \in X$. The sections s on U and t on V are called *germ-equivalent* at x, written $s \sim_x t$, if there exists an (open) neighbourhood $W \subseteq U \cap V$ of x such that the restrictions of s and t to W are equal, i.e. $res_{W,U}(s) = res_{W,V}(t)$. A *germ* at x is an germ-equivalence class of sections, denoted $[s]_x$, i.e. $[s]_x = \{\sigma | \sigma \sim_x s\}$. The set of germ-equivalence classes is the stalk $\mathcal{F}_x$.

Remark 94. Germs, like stalks, are defined by universal construction, i.e. colimits. The equivalence classes for an equivalence relation R on a set A are determined by coequalizer of projections, $R \rightrightarrows A$, i.e. the pair $(A/R, q)$, where A/R is the set of classes $q : A \to A/R$ assigns to each element its equivalence class.

Example 95 (suits)**.** The germs of suit are the equivalence classes of cards with the same suit: e.g., $[\heartsuit] = \{2\heartsuit, \ldots, A\heartsuit\}$.

Definition 96 (presheaf/sheaf morphism)**.** Suppose $\mathcal{F}, \mathcal{G} : X^{\mathrm{op}} \to \mathbf{Set}$ are presheaves (sheaves). A *presheaf (sheaf) morphism* $\eta : \mathcal{F} \to \mathcal{G}$ is a family of maps $\eta = \{\eta_U : \mathcal{F}(U) \to \mathcal{G}(U)\}_{U \in \mathcal{O}_X}$ such that the following diagram commutes:

$$\begin{array}{ccc} \mathcal{F}(U) & \xrightarrow{\eta_U} & \mathcal{G}(U) \\ {\scriptstyle res_{V,U}}\downarrow & & \downarrow{\scriptstyle res_{V,U}} \\ \mathcal{F}(V) & \xrightarrow[\eta_V]{} & \mathcal{G}(V) \end{array} \tag{S30}$$

for each inclusion $V \subseteq U$ of X, i.e. a natural transformation.

Example 97 (inclusion of presheaves)**.** An inclusion of presheaves, written $\mathcal{F} \subseteq \mathcal{G}$, is a presheaf morphism such that the component at each open set U of X is an injection of sections, $\iota_U : \mathcal{F}(U) \subseteq \mathcal{G}(U)$. $\mathcal{F}$ is called a *sub(pre)sheaf* of $\mathcal{G}$.

Remark 98. The category of presheaves on a topological space X is a functor category, denoted $\mathbf{Psh}(X)$ or $\mathbf{Set}^{X^{\mathrm{op}}}$. Likewise, the category of sheaves on X is also a functor category, denoted $\mathbf{Sh}(X)$.

Definition 99 (sheafification)**.** The *sheafification functor* $(-)^+ : \mathbf{Psh}(X) \to \mathbf{Sh}(X)$ sends every presheaf $\mathcal{F}$ to its "nearest" sheaf, denoted $\mathcal{F}^+$ as the universal construction given by the following commutative diagram:

$$\begin{array}{ccccc} \mathcal{F} & \xrightarrow{\sigma} & \mathcal{F}^+ & \qquad & \mathcal{F}^+ \\ & {\scriptstyle\phi}\searrow & \downarrow{\scriptstyle u} & & \downarrow{\scriptstyle u} \\ & & \mathcal{G} & & \mathcal{G} \end{array} \tag{S31}$$

Sheafification is left adjoint to inclusion, $\mathbf{Sh}(X) \subseteq \mathbf{Psh}(X)$. $\mathbf{Sh}(X)$ is a subcategory of $\mathbf{Psh}(X)$.

Remark 100. Sheafification acts like completion by adding the missing rows of the corresponding table.

Definition 101 (direct image functor)**.** Suppose X and Y are topological spaces and $f : X \to Y$ is a continuous function. The *direct image functor* along f, written $f_* : \mathbf{Psh}(X) \to \mathbf{Psh}(Y)$, sends each presheaf $\mathcal{F}$ to the presheaf $f_*\mathcal{F} : U \mapsto \mathcal{F}(f^{-1}[U])$, i.e. the sections of $f_*\mathcal{F}$ over an open set U are the sections of $\mathcal{F}$ over the preimage of U under f.

Remark 102. The direct image functor for sheaves is the same as for presheaves.

Example 103 (iconization)**.** The direct image functor acts like a "chunking" process when the $f : X \to Y$ is a constant function, sending each element $x \in X$ to the same value in Y. For instance, suppose a map sending suit and rank to the only element in the singleton set $1 = \{*\}$. The resulting sheaf is just the set of cards without access to the suit and ranks, i.e. a set of 52 (distinct) icons.

Definition 104 (inverse image functor)**.** Suppose topological spaces X and Y, and continuous function $f : X \to Y$. The *inverse image functor* for presheaves back along f, written $f^* : \mathbf{Psh}(Y) \to \mathbf{Psh}(X)$, is the presheaf $\mathcal{G}$ on Y defined as $f^*\mathcal{G}(U) := \lim_{f[U] \subseteq V} \mathcal{G}(V)$ for each open set U of Y, i.e. the sections are given by the colimit over the open sets containing the image of U.

Remark 105. The inverse image functor for sheaves is obtained by sheafifying the presheaf obtained from the inverse image functor.

Example 106 (discretization)**.** The inverse image functor acts like a "dechunking" process for $f : X \to 1$, i.e. the resulting sheaf has the sections on the only element in $\{*\}$ as the sections on each element in X—cf. diagonal functor, $\Delta : X \mapsto (X, X)$. In other words, a single dimension is split into two dimensions, which affords compositional (symbolic) representations of icons, e.g., to recover the suit and rank dimensions for the set of cards from their icons.

Remark 107. The inverse and direct image functors for sheaves are adjoints, $f^* \dashv f_*$. For the continuous function on the two-point (discrete) space, $f : 2 \to 1$, i.e. an adjoint situation $\mathbf{Sh}(2) \rightharpoonup \mathbf{Sh}(1)$, cf. $\Delta \dashv \Pi$.

2.4 Bundles

Definition 108 (fibre bundle)**.** A *fibre bundle* is a 4-tuple (E, B, π, F) consisting of a topological space E, called the *total space*, a topological space B, called the *base space*, a continuous surjective function $\pi : E \to B$, called the *projection map*, and a topological space F, called the *fibre*, that together satisfy

- local triviality: diagram

$$
\begin{array}{ccc}
\pi^{-1}[U] & \xrightarrow{\ \phi\ } & U \times F \\
{\scriptstyle \pi}\big\downarrow & \swarrow {\scriptstyle \pi_U} & \\
U & &
\end{array}
\tag{S32}
$$

commutes for every open set U of B, where π_U is the natural projection.

Remark 109. The map ϕ specifies the extent to which a local product space is a global product space.

Example 110 (trivial bundle)**.** The fibre bundle $(B \times F, B, \pi_B, F)$ is called a *trivial bundle*. (π_B is the natural projection.) In this case, ϕ is just an identity map.

Remark 111. The trivial bundle highlights one way to think about fibre bundles as a subset of a product of elements in a base, $\{b, c, d\}$, and elements in a fibre, $\{f, g, h\}$:

	(c, f)	(d, f)		(b, f)	(c, f)	(d, f)
(b, g)	(c, g)		$\subseteq$	(b, g)	(c, g)	(d, g)
(b, h)		(d, h)		(b, h)	(c, h)	(d, h)

Example 112 (regular table)**.** A (regular) table corresponds to a trivial bundle (Table S1). The base space is the set of column names $X = \{A, B\}$ with the discrete topology, the fibre is the set of row identifiers, $F = \{1, 2, 3\}$ also with the discrete topology, the total space is the product space, $X \times F$, and the projection map is the natural projection sending each set of cells to the column names to which they belong, $\pi_X : X \times F \to X$, e.g., $\pi_{\{A\}} : (A, i) \mapsto A$ and $\pi_{\{A,B\}} : (\{A, B\}, j) \mapsto \{A, B\}$.

1	A_1	B_1
2	A_2	B_2
3	A_3	B_3
	A	B

Table S1. A (regular) table as a trivial fibre bundle.

Example 113 (ragged table)**.** A ragged table (i.e. where columns have different lengths) is an example of a non-trivial fibre bundle (Table S2). In this situation, the third row does not have a cell in the B column. ϕ acts as an injection (but not an identity map) of the cells into the complete table in example 112. Thus, the bundle is locally but not globally a product space.

1	A_1	B_1
2	A_2	B_2
3	A_3	
	A	B

Table S2. A ragged table as a non-trivial fibre bundle.

Definition 114 (étalé space)**.** Suppose a sheaf $\mathcal{F}$ in $\mathbf{Sh}(X)$. The *étalé space* of $\mathcal{F}$ is a topological space E together with a projection $\pi : E \to X$ such that the sheaf of sections of π is $\mathcal{F}$. Here, the sheaf of sections is written $\Gamma(\pi, -)$.

Remark 115. In detail, the étalé space is constructed from the disjoint union of the stalks $\mathcal{F}_x$ of $\mathcal{F}$, i.e. $\bigcup_{x \in X} \mathcal{F}_x = \{(x, s) | x \in X, s \in \mathcal{F}_x\}$. The topological space E is defined as follows.

- The points of E are the pairs $(x, [s]_x)$ that are determined for each open set U of X, i.e. the germs $[s]_x$ of the sections $s \in \mathcal{F}(U)$ for each x of U.
- Each open set of the topology for E is declared to be the (set) union of the pairs $(x, [s]_x)$ for each x in U and $s \in \mathcal{F}(U)$, i.e. the set $\bigcup_{x \in U}(x, [s]_x)$.

The projection $\pi : E \to X$ is $\pi(x, [s]_x) = x$.

Remark 116. The category of étalé spaces over X is equivalent to the category of sheaves on X.

Definition 117 (bundle morphism). Suppose $\mathcal{E} = (E, B, \pi, F)$ and $\mathcal{E}' = (E', B', \pi', F')$ are fibre bundles. A *bundle morphism* from $\mathcal{E}'$ to $\mathcal{E}$ is a pair of continuous maps $(\phi : E' \to E, f : B' \to B)$ such that $\pi \circ \phi = f \circ \pi'$, as indicated by the following commutative diagram:

$$
\begin{array}{ccc}
E' & \xrightarrow{\phi} & E \\
{\scriptstyle \pi'}\downarrow & & \downarrow{\scriptstyle \pi} \\
B' & \xrightarrow[f]{} & B
\end{array}
\tag{S33}
$$

Remark 118. A bundle morphism preserves projections relative to the change in base space.

Definition 119 (pullback bundle). Suppose $\mathcal{E} = (E, B, \pi, F)$ is a fibre bundle and $f : B' \to B$ is a continuous map. The *pullback bundle* (for $\mathcal{E}$ and f) is defined as the fibre bundle (f^*E, B', π', E) where

- the set $f^*E = \{(b', e) \in B' \times E | f(b') = \pi(e)\}$ has the subspace topology,
- the projection map $\pi' : f^*E \to B'$ is the (first) natural projection $\pi'(b', e) = b'$ and
- $h : f^*E \to E$ is the (second) natural projection, i.e. $h : (b', e) \to e$, such that $\pi \circ h = f \circ \pi'$, as indicated by commutative diagram

$$
\begin{array}{ccc}
f^*E & \xrightarrow{h} & E \\
{\scriptstyle \pi'}\downarrow & & \downarrow{\scriptstyle \pi} \\
B' & \xrightarrow[f]{} & B
\end{array}
\tag{S34}
$$

The bundle f^*E is called the *pullback of* E *by* f, or the *bundle induced by* f.

Remark 120. A pullback bundle is a fibre bundle, so the pair of maps (h, f) is a bundle morphism.

2.5 Topoi

Definition 121 (subobject). In a category $\mathbf{C}$, a *subobject* of an object C is an equivalence class of monomorphisms $[i] = \{j : B \to C | j \sim i : A \to C\}$, where $j \sim i$ if there exists an isomorphism $k : A \to B$ such that $i = j \circ k$, as shown by commutative diagram

$$
\begin{array}{ccc}
A & \xrightarrow{\quad k \quad} & B \\
& {\scriptstyle i}\searrow \quad \swarrow{\scriptstyle j} & \\
& C &
\end{array}
\tag{S35}
$$

Example 122 (subset)**.** A subobject in **Set** is a subset.

Remark 123. There is a dual concept called *quotient object*.

Definition 124 (subfunctor)**.** A *subfunctor* of a functor $G : \mathbf{C} \to \mathbf{D}$ is a subobject in the category of functors $\mathbf{D}^{\mathbf{C}}$, i.e. a pair (F, i) consisting of

- a functor $F : \mathbf{C} \to \mathbf{D}$ and
- a natural monomorphism $i : F \dot{\to} G$, i.e. every component $i_A : F(A) \to G(A)$ is a monomorphism.

As a subobject the subfunctor is the equivalence class of natural monomorphisms, $[i]$.

Example 125 (subpresheaf)**.** Suppose a (set-valued) presheaf $G : \mathbf{C}^{\mathrm{op}} \to \mathbf{Set}$. A *subpresheaf* of G is a presheaf $F : \mathbf{C}^{\mathrm{op}} \to \mathbf{Set}$ such that

- $F(C) \subseteq G(C)$ for every object C in $\mathbf{C}$ and
- $F(f) = G(f)|_{F(C)}$ for every morphism $f : C' \to C$ in $\mathbf{C}$, i.e. $G(f)$ restricted to $F(C)$.

A subpresheaf F of G is written $F \subseteq G$.

Example 126 (subsets)**.** When $\mathbf{C}$ is the terminal category $\mathbf{1}$, the subfunctors $F : \mathbf{1} \to \mathbf{Set}$ correspond to subsets: the functor $S : \mathbf{1} \to \mathbf{Set}$ picks out the set S and its identity arrow 1_S and the subfunctor $U \subseteq S$ picks out the subset $U \subseteq S$ and the identity arrow $1_U = 1_S|_U$.

Definition 127 (subobject classifier)**.** In a category $\mathbf{C}$ with finite limits, a *subobject classifier* is a monomorphism $t : 1 \to \Omega$ from the terminal object to an object Ω, called the *classifying object* such that for every monomorphism $m : U \to X$ the exists a unique arrow $\chi_U : X \to \Omega$ such that the following diagram is a pullback:

$$\begin{array}{ccc} U & \xrightarrow{\;!\;} & 1 \\ {\scriptstyle m}\big\downarrow & & \big\downarrow{\scriptstyle t} \\ X & \xrightarrow[\chi_U]{} & \Omega \end{array} \tag{S36}$$

Object Ω is also called the *truth object*.

Remark 128. A subobject is a kind of universal construction, hence a pair (Ω, t) consisting of the classifying object, Ω, and the monomorphism $t : 1 \to \Omega$. As such, the classifying object acts like a terminal object in the category of monomorphisms, noting that ! is unique: for every pair (X, m) there exists a unique arrow to the classifying object as indicated by the following diagram:

$$(X, m) \dashrightarrow (\Omega, t) \tag{S37}$$

where the morphism is the pair of arrows $(\chi_U, !)$.

Example 129 (relations)**.** Suppose a binary relation $R \subseteq A \times B$. The characteristic function $\chi_R : (a, b) \mapsto 1$ if $(a, b) \in R$, or 0 if $(a, b) \notin R$ is a subobject classifier, indicated by the pullback diagram

$$\begin{array}{ccc} R & \xrightarrow{\;!\;} & 1 \\ {\scriptstyle \subseteq}\big\downarrow & & \big\downarrow{\scriptstyle T} \\ A \times B & \xrightarrow[\chi_R]{} & \mathbb{B} \end{array} \tag{S38}$$

where $\mathbb{B} = \{0, 1\}$ and $T : * \mapsto 1$ picks out the boolean value 1 corresponding to True.

Remark 130. The correspondence between subobject and characteristic map affords translation between extensional vs. intentional specification of a relation that exploits a trade-off: small relations may be more easily expressed by spelling out each instance, whereas larger relations by defining the map.

Example 131 (subfunctions)**.** A notion of "subfunction" of a function $f : X \to Y$ can be given as a subobject g obtained from the following pullback:

$$\begin{array}{ccc} g & \xrightarrow{!} & 1 \\ {\scriptstyle\subseteq}\downarrow & & \downarrow{\scriptstyle T} \\ f & \xrightarrow[\chi_g]{} & \mathbb{B} \end{array} \tag{S39}$$

where f and g are understood as the graphs (relations) $\Gamma(f)$ and $\Gamma(g)$, respectively. A subfunction *as a function* can be given by working in the category of arrows in the category of sets and functions, i.e. $\mathbf{Arr}(\mathbf{Set})$ whose objects are functions—the subobjects in this category are the (equivalence classes of) subfunctions (see definition 121).

Remarks 132. A collection of subobjects constitutes a partially ordered set (poset), which affords a categorical notion of incremental (partial) learning as the subfunctions of a target function, f_{tar}, in the context of the available resources and system goals.

1. The collection of subfunctions forms a poset, $(\Phi, \subseteq)$ with f_{tar} as the top element. Suppose a (monotonic) error function $Err : (\Phi, \subseteq) \to \mathbb{R}^{\text{op}}$ assigns to each function $f \in \Phi$ an error or cost in the set of real numbers with the order $x \geq y$. Learning (training) can be expressed as a universal morphism from Err to 0, as indicated by commutative diagram

$$\begin{array}{ccccc} f & \qquad & Err(f) & & \\ {\scriptstyle\subseteq}\,\vdots & & {\scriptstyle\geq}\,\vdots & \searrow^{\geq} & \\ f_{train} & & E_{min} & \xrightarrow[\geq]{} & 0 \end{array} \tag{S40}$$

   i.e. the function f_{train} obtained as the minimum error, E_{min}, for the given error (cost) function.
2. As a universal morphism from functor Err to object 0, a (sub)functor $Err' \subseteq Err$ acts as a (limited) resource and an object $E_{err'} \geq 0$ acts as a (sub)goal. The subgoal is to reduce error to $E_{err'}$ given the resources afforded by a subcategory of $(\Phi, \subseteq)$, affording limited information about input-output relationships of the target function.

Example 133 (subsheaf)**.** Suppose a topological space X and a sheaf $\mathcal{F}$ on X. The subobject classifier for subsheaves of $\mathcal{F}$, i.e. the natural transformations $j : \mathcal{G} \to \mathcal{F}$ is given by the pullback

$$\begin{array}{ccc} \mathcal{G} & \xrightarrow{!} & 1 \\ {\scriptstyle j}\downarrow & & \downarrow{\scriptstyle \eta} \\ \mathcal{F} & \xrightarrow[\chi_j]{} & \Omega \end{array} \tag{S41}$$

which consists of the following data:

- the terminal sheaf, $1 : U \mapsto \{*\}$ that assigns to each open set U of X the one-point set $\{*\}$,
- the classifying object (sheaf) $\Omega : U \mapsto \mathcal{O}_X(U)$ that assigns to each open set U of X the set of open sets of U, i.e. $\mathcal{O}_X(U)$, and to each inclusion $V \subseteq U$ the restriction $res_{V,U} : \mathcal{O}_X(U) \to \mathcal{O}_X(V)$,
- the monomorphism (natural transformation) η consisting of the family of maps $\eta_U : 1(U) \to \Omega(U)$ that sends the only section $*$ on U to the open set U, i.e. for each inclusion $V \subseteq U$ of X the following diagram commutes:

$$\begin{array}{ccccc} U & & 1(U) = \{*\} & \xrightarrow{\eta_U} & \mathcal{O}_X(U) = \Omega(U) \\ {\scriptstyle\subseteq}\uparrow & & {\scriptstyle res_{V,U}=1}\downarrow & & \downarrow{\scriptstyle res_{V,U}} \\ V & & 1(V) = \{*\} & \xrightarrow[\eta_V]{} & \mathcal{O}_X(V) = \Omega(V) \end{array} \tag{S42}$$

where $\eta_U : * \mapsto U$ and $\eta_V : * \mapsto V$,
- a subsheaf $j : \mathcal{G} \to \mathcal{F}$,
- a morphism $! : \mathcal{G} \to \mathcal{F}$ consisting of the family of maps $!_U : \mathcal{G}(U) \to 1(U)$ that sends each section s of $\mathcal{G}(U)$ to the only section $*$ of $1(U)$, i.e. for each inclusion $V \subseteq U$ of X the following diagram commutes:

$$\begin{array}{ccccc} U & & \mathcal{G}(U) & \xrightarrow{!_U} & 1(U) = \{*\} \\ {\scriptstyle\subseteq}\uparrow & & {\scriptstyle res_{V,U}}\downarrow & & \downarrow{\scriptstyle res_{V,U}=1} \\ V & & \mathcal{G}(V) & \xrightarrow[!_V]{} & 1(V) = \{*\} \end{array} \tag{S43}$$

where $!_U : s \mapsto *$ and $!_V : t \mapsto *$ and
- a classifying morphism χ_j consisting of the family of maps $\chi_{j,U} : \mathcal{F}(U) \to \Omega(U)$ that sends each section s of $\mathcal{F}(U)$ to the union of open sets V of U such that the restriction of s to V is contained in the sections $j_V(G(V))$, i.e. for each inclusion $V \subseteq U$ of X the following diagram commutes:

$$\begin{array}{ccccc} U & & \mathcal{F}(U) & \xrightarrow{\chi_{j,U}} & \Omega(U) \\ {\scriptstyle\subseteq}\uparrow & & {\scriptstyle res_{V,U}}\downarrow & & \downarrow{\scriptstyle res_{V,U}} \\ V & & \mathcal{F}(V) & \xrightarrow[\chi_{j,V}]{} & \Omega(V) \end{array} \tag{S44}$$

where $\chi_{j,U} : s \mapsto \bigcup_i V_i$ such that $V_i \subseteq U$ and $s \in j(G(V_i))$ and $\chi_{j,V} : t \mapsto \bigcup_k W_k$ such that $W_k \subseteq V$.

Remark 134. The subobject classifier for sheaves says to what extent is a section of $\mathcal{F}$ a section of $\mathcal{G}$, where extent is "measured" by the open sets of X. If a global section s of $\mathcal{F}$ is a global section of $\mathcal{G}$, then the subobject classifier maps s to X, otherwise to some open subset of X, which is the empty set when no part (restriction) of s is a section of $\mathcal{G}$. A concrete example of a sheaf as a relational database table illustrates this situation. Suppose a discrete topology for the set $X = \{A, B\}$ and a subsheaf $j : \mathcal{G} \to \mathcal{F}$, where $\mathcal{G}$ is given by the relational table

a_1	b_2
a_1	b_1
A	B

and $\mathcal{F}$ by the relational table

a_2	b_3
a_2	b_2
a_2	b_1
a_1	b_3
a_1	b_2
a_1	b_1
A	B

Example actions of the subobject classifier χ_j on a section of $\mathcal{F}$ follow:

- $\chi_{j,X} : a_1b_1 \mapsto \{A, B\}$, i.e. $a_1b_1|_A = a_1 \in j_A(\mathcal{G}(A)) = \{a_1\}$ and $a_1b_1|_B = b_1 \in j_B(\mathcal{G}(B)) = \{b_1, b_2\}$
- $\chi_{j,X} : a_1b_3 \mapsto \{A\}$, i.e. $a_1b_3|_A = a_1 \in j_A(\mathcal{G}(A)) = \{a_1\}$ and $a_1b_3|_B = b_3 \notin j_B(\mathcal{G}(B)) = \{b_1, b_2\}$
- $\chi_{j,X} : a_1b_3 \mapsto \emptyset$, i.e. $a_2b_3|_A = a_2 \notin j_A(\mathcal{G}(A)) = \{a_1\}$ and $a_2b_3|_B = b_3 \notin j_B(\mathcal{G}(B)) = \{b_1, b_2\}$
- $\chi_{j,A} : a_1 \mapsto \{A\}$, i.e. $a_1|_A = a_1 \in j_A(\mathcal{G}(A)) = \{a_1\}$
- $\chi_{j,A} : a_2 \mapsto \emptyset$, i.e. $a_2|_A = a_2 \notin j_A(\mathcal{G}(A)) = \{a_1\}$.

Remark 135. The subobject classifier for subpresheaves of a presheaf is analogous to that for sheaves.

Definition 136 (elementary topos)**.** A *elementary topos* is a category that has

- finite limits and
- exponential objects.

Remark 137. All finite limits and exponential objects implies all finite colimits and a subobject classifier.

Examples 138 (topoi)**.** Some examples of topoi (toposes) follow.

- The categories of presheaves on a topological space X, i.e. $\mathbf{Psh}(X)$, is a topos; likewise for sheaves.
- $\mathbf{Set} \cong \mathbf{Psh}(1)$.
- The category of fibre bundles over X, i.e. $\mathbf{Bund}/X$, is a topos; likewise for étalé spaces.

Remark 139. The existential ($\exists x$) and universal ($\forall x$) quantifiers can be expressed as the left and right adjoints (respectively) of the inverse image functor. In regard to sets, suppose a function $f : X \to Y$, so $\mathcal{P}(X)$ and $\mathcal{P}(Y)$ are categories of sets and inclusions and f is a continuous map. The *inverse image functor* induced by f is the functor $f^* : \mathcal{P}(Y) \to \mathcal{P}(X)$ that sends each subset U of Y to the preimage $f^{-1}[U] = \{x|f(x) \in U\}$ and each inclusion $U \subseteq V$ to the inclusion $f^{-1}[U] \subseteq f^{-1}[V]$.

- The *existential quantifier functor* $\exists_f : \mathcal{P}(X) \to \mathcal{P}(Y)$ sends each subset U of X to the image of U under f, i.e. the set $\exists_f(U) = \{y \in Y|f(x) = y, x \in U\}$, and inclusion $U \subseteq V$ to the inclusion $f[U] \subseteq f[V]$.
- The *universal quantifier functor* $\forall_f : \mathcal{P}(X) \to \mathcal{P}(Y)$ sends each subset U of X to the set of elements $y \in Y$ whose preimages are contained in U, i.e. the set $\forall_f(U) = \{y \in Y|f^{-1}[y] \subseteq U\}$, and inclusion $U \subseteq V$ to the inclusion $f[U] \subseteq f[V]$.

We have adjoint situations $\exists_f \dashv f^*$ and $f^* \dashv \forall_f$.

3 A LOT IN A NUTSHELL

A LoT is framed by categorical structure pertaining to spaces whose topology gives "shape" to the associated data in the form of percepts and concepts, as illustrated by the diagram

$$\begin{array}{ccc} \text{percept} & \longrightarrow & \text{percept}' \\ \uparrow\dashv\downarrow & & \uparrow\dashv\downarrow \\ \text{concept} & \longrightarrow & \text{concept}' \end{array} \tag{S45}$$

(informally) depicting the several kinds of arrows:

- vertical (upwards): perceptualization of concepts as presheaves/sheaves (percepts) on a topological space X (concepts), i.e. the functors $\mathcal{F} : X^{\mathrm{op}} \to \mathbf{Set}$,
- vertical (downwards): conceptualization of percepts as fibre bundles $\mathcal{E} = (E, X, \pi, F)$ over X,
- horizontal (top): perceptual change as presheaf or bundle morphisms,
- horizontal (bottom): conceptual change has maps between (conceptual) spaces, and
- duals ($\dashv$): dualization of perception/conception as adjoint functors between the categories of presheaves/bundles on/over X.

The total space E of a fibre bundle is also a topological space, so percepts (like concepts) have shape. Continuous functions between topological spaces underpin the connection between discrete (symbolic) and non-discrete (non-symbolic) representations. In this way, LoT connects to other representational formats both "vertically" between percepts and concepts and "horizontally" between symbolic and non-symbolic representational systems.

3.1 What is an object file, categorically?

An object file is supposed to be a (universal) duality between a fibre bundle and a presheaf: a fibre bundle is a map from a percept to a concept of an object; dually, a presheaf is a map from the concept to a percept of the object. Maps between bundles and maps between presheaves corresponds to changes in perception and conception of the object (diagram S45). Conceptualization is a projection from a percept to a concept and perceptualization is a presheaf from a concept to a percept.

Object representations are composed from information regarded as the role that some filler plays in a particular context. In terms of presheaves, the global sections of a presheaf represent the context for the data (sections) that fill the role given by the open sets. Suppose, for instance, a visual field consisting of a *red triangle*, a *blue square* and a *red square*. This situation is expressed as a presheaf on the topological space $X = \{C, S, L\}$ for colour, shape and location with the topology $T = \{\emptyset, \{L\}, \{L, C\}, \{L, S\}, X\}$ and global sections given by the triples $o_1 = (\mathrm{red}, \triangle, l_1)$, $o_2 = (\mathrm{blue}, \square, l_2)$ and $o_3 = (\mathrm{red}, \square, l_3)$. Each point (open set) in the space corresponds to a feature dimension (role) and each section for an open set to the corresponding features (fillers). Both situations are also sheaves as the data can be recovered from the stalks, i.e. the feature maps. In database terms, the data are essentially recovered by joining the tables corresponding to the sections on the open sets containing colour and shape: the pairs (red, l_1), (blue, l_2), (red, l_3) and the pairs $(\triangle, l_1)$, $(\square, l_2)$, $(\triangle, l_3)$ are joined along the common locations. This situation extends to additional feature dimensions, such as frequency: a presheaf on the topological space $X = \{C, S, F, L\}$ with topology $T = \{\emptyset, \{L\}, \{L, C\}, \{L, S\}, \{L, F\}, X\}$ as shown for a visual search task (Phillips et al., 2012, Text S3). Dually, in terms of fibre bundles, the projection map sends that data for each coloured shape in the visual

field to features dimensions, e.g., $\pi : ((o_1, \mathrm{red}), C) \mapsto C, ((o_1, \triangle), S) \mapsto S$, which act as the conceptual roles. The difference between bundles and presheaves, in database terms, is analogous to the difference between rows-first vs. columns-first view of tables, i.e. rows correspond to global sections and columns correspond to fibres. The category of presheaves on a topological space, X, is equivalent to the category of fibre bundles over X, i.e. $\mathbf{Psh}(X) \simeq \mathbf{Bund}/X$. This relationship affords recovery of a percept from an occlusion event: the map from percept to concept (bundle) is sent to the corresponding presheaf which is a map from the concept back to a percept.

REFERENCES

Abramsky, S. (2013). Relational databases and Bell's theorem. In *In Search of Elegance in the Theory and Practice of Computation*, eds. V. Tannen, L. Wong, L. Libkin, W. Fan, W. C. Tan, and M. Fourman (Berlin: Springer-Verlag), vol. 8000 of *Lecture Notes in Computer Science*, chap. 2. 13–35

Abramsky, S. and Brandenburger, A. (2011). The sheaf-theoretic structure of non-locality and contextuality. *New Journal of Physics* 13, 113036. doi:10.1088/1367-2630/13/11/113036

Awodey, S. (2010). *Category theory*. Oxford Logic Guides (New York, NY: Oxford University Press), 2nd edn. doi:10.5555/2060081

Goldblatt, R. (2006). *Topoi: The categorial analysis of logic* (New York, NY: Dover Publications), revised edn.

Hartshorne, R. (2013). *Algebraic geometry*, vol. 52 of *Graduate Texts in Mathematics* (New York, NY: Springer-Verlag). doi:10.1007/978-1-4757-3849-0

Lawvere, F. W. (1991). *Some thoughts on the future of category theory* (Berlin, Germany: Springer-Verlag), vol. 1488 of *Lecture Notes in Mathematics*. 1–13

Lawvere, F. W. and Schanuel, S. H. (2009). *Conceptual mathematics: A first introduction to categories* (Cambridge, UK: Cambridge University Press). doi:10.1017/CBO9780511804199

Leinster, T. (2014). *Basic category theory*, vol. 143 of *Cambridge Studies in Advanced Mathematics* (Cambridge, UK: Cambridge University Press). doi:10.1017/CBO9781107360068

Mac Lane, S. (1992). The protean character of mathematics. In *The Space of Mathematics*, eds. J. Echieverria, A. Ibarra, and T. Mormann (Berlin: de Gruyter). 3–13

Mac Lane, S. (1997). Categorical foundations of the protean character of mathematics. In *Philosophy of Mathematics Today*, eds. E. Agazzi and G. Darvas (Dordrecht: Kluwer Academic). 117–122

Mac Lane, S. (1998). *Categories for the working mathematician*. Graduate Texts in Mathematics (New York, NY: Springer), 2nd edn. doi:10.1007/978-1-4757-4721-8

Mac Lane, S. and Moerdijk, I. (1992). *Sheaves in geometry and logic: A first introduction to topos theory*. Graduate Texts in Mathematics (New York, NY: Springer). doi:10.1007/978-1-4612-0927-0

Phillips, S., Takeda, Y., and Singh, A. (2012). Visual feature integration indicated by phase-locked frontal-parietal EEG signals. *PLoS One* 7, e32502. doi:10.1371/journal.pone.0032502

Spivak, D. I. (2014). *Category theory for the sciences* (Cambridge, MA: MIT Press). doi:10.5555/2628001
